# pathCHEMO, a generalizable computational framework uncovers molecular pathways of chemoresistance in lung adenocarcinoma

**DOI:** 10.1038/s42003-019-0572-6

**Published:** 2019-09-06

**Authors:** Nusrat J. Epsi, Sukanya Panja, Sharon R. Pine, Antonina Mitrofanova

**Affiliations:** 1 Department of Health Informatics, Rutgers School of Health Professions, Rutgers Biomedical and Health Sciences, Newark, NJ 07107 USA; 2 Departments of Pharmacology and Medicine, Rutgers Cancer Institute of New Jersey, Robert Wood Johnson Medical School, New Brunswick, NJ 08901 USA; 3 Rutgers Cancer Institute of New Jersey, Rutgers, The State University of New Jersey, New Brunswick, NJ 08901 USA

**Keywords:** Computational models, Predictive medicine, Lung cancer

## Abstract

Despite recent advances in discovering a wide array of novel chemotherapy agents, identification of patients with poor and favorable chemotherapy response prior to treatment administration remains a major challenge in clinical oncology. To tackle this challenge, we present a generalizable genome-wide computational framework pathCHEMO that uncovers interplay between transcriptomic and epigenomic mechanisms altered in biological pathways that govern chemotherapy response in cancer patients. Our approach is tested on patients with lung adenocarcinoma who received adjuvant standard-of-care doublet chemotherapy (i.e., carboplatin-paclitaxel), identifying seven molecular pathway markers of primary treatment response and demonstrating their ability to predict patients at risk of carboplatin-paclitaxel resistance in an independent patient cohort (log-rank *p*-value = 0.008, HR = 10). Furthermore, we extend our method to additional chemotherapy-regimens and cancer types to demonstrate its accuracy and generalizability. We propose that our model can be utilized to prioritize patients for specific chemotherapy-regimens as a part of treatment planning.

## Abstract (web summary)

Nusrat Epsi et al. present pathCHEMO, a computational framework for uncovering transcriptomic and epigenomic pathways of chemoresistance in cancer that has the potential to improve clinical decision-making. They apply pathCHEMO to lung adenocarcinoma data from public databases, and identify seven molecular pathways implicated in carboplatin-paclitaxel resistance.

## Introduction

Lung adenocarcinoma (LUAD) is a major cause of cancer-related death in the United States with a five-year survival rate of 17.7%^1^. The majority of patients with LUAD lack clinically actionable mutations and are commonly administered a doublet chemotherapy (i.e., platinum-based chemotherapy often combined with plant alkaloids and/or antimetabolites) to improve response rates and survival^2–5^. Most recently, treatment for LUAD has also included immune checkpoint inhibitors, yet they are not curative for most patients^6^. The heterogeneity of response to the standard-of-care therapies and rapidly emerging treatment resistance remain major challenges in lung cancer management. Prioritization of patients based on their risk of developing resistance prior to therapy administration would improve disease course and enhance informed clinical decision making at large.

In recent years, several successful attempts^7–12^ have improved classification of LUAD based on markers of overall disease aggressiveness, including mutations in oncogenes (*EGFR*^7^, *KRAS*^8^), proto-oncogenes (*ERBB2*^9^, *BRAF*^10^), and tumor suppressor genes (*TP53*^11^, *PTEN*^12^). Despite being successful as prognostic markers of LUAD aggressiveness, they have not been associated with the complexity of therapeutic response yet^13,14^, suggesting that more complex mechanisms might be at play in this malignancy.

Recently, multiple transcriptomic and epigenomic alterations have been highlighted to play a role in primary and secondary chemoresistance across various cancer types^15–20^. For example, studies focused on transcriptomic alterations have demonstrated that: *MDR1* amplification is implicated in acquired resistance to anthracyclines, vinca alkaloids, and other antineoplastic chemotherapies in breast cancer^15^; over-expression of dihydrodiol dehydrogenase enzyme is central in resistance to cisplatin in ovarian cancer^16^; and higher genomic instability due to p53 inactivation is essential in resistance to platinum-based chemotherapy in ovarian cancer^17^. In parallel, epigenomic-centered studies have demonstrated that: genome-wide hypermethylation is implicated in resistance to antineoplastic fotemustine in melanoma^18^; hypermethylation of *DKK3* leads to docetaxel resistance in non-small cell lung cancer^19^; and hypomethylation of *MIR663A* induce cyclophosphamide and docetaxel resistance in breast cancer^20^. Given the success of individual transcriptomic and epigenomic determinants of chemoresponse, a systematic genome-wide investigation of the interplay between transcriptomic and epigenomic mechanisms implicated in resistance can provide valuable predictive markers of predisposition to chemotherapy failure.

In the past decade, several computational methods have been successfully applied to understand cancer initiation and progression through integration of transcriptomic and epigenomic data, including correlation of mRNA expression and DNA methylation and/or copy number variations^21–23^, linear regression connecting DNA methylation sites and mRNA expression of the site-harboring genes^24^, network-based integration of mRNA expression and DNA methylation and/or copy number variations^25–27^. Even though successful in identifying clinically relevant signatures of disease progression, these methods have not yet fully explored the interplay between transcriptomic and epigenomic mechanisms altered in molecular pathways implicated in chemo response, which would shed light on complex molecular mechanisms that govern therapeutic resistance.

In this work, we develop a generalizable computational framework to identify molecular pathways altered on transcriptomic (i.e., mRNA expression) and epigenomic (i.e., DNA methylation) levels that govern resistance to chemotherapy. We name our approach pathCHEMO—uncovering transcriptomic and epigenomic pathways implicated in CHEMOresistance. Our overall idea is that pathways that are altered on both mRNA expression and DNA methylation levels are more likely to capture complex relationships implicated in therapeutic resistance and overcome noise present in any single experiment or data type. In addition, our approach provides several important advantages that tackle complexity of treatment response. First, it uncovers molecular pathways altered on both transcriptomic and epigenomic levels, which increases the likelihood to identify functionally relevant alterations. Second, these pathways can be utilized as effective markers of primary chemoresistance to predict patients with poor and favorable response, even prior to therapy administration. Finally, it uncovers molecular pathways, rather than single determinants, thus providing potential functional candidates for therapeutic intervention to preclude or overcome resistance. Motivated by the need for markers of chemoresponse in lung cancer, we analyze profiles of patients with LUAD from The Cancer Genome Atlas (TCGA-LUAD)^28^, which received adjuvant standard-of-care chemotherapy (i.e., a combination of platinum-based carboplatin and plant alkaloid paclitaxel). pathCHEMO identifies seven molecular pathways altered on transcriptomic and epigenomic levels that differentiate patients with poor and favorable carboplatin–paclitaxel response. We demonstrate that the activity of these pathways as well as their representative read-out genes, can serve as molecular markers to identify patients at risk of resistance to carboplatin–paclitaxel in an independent patient cohort^5^ (log-rank *p*-value = 0.0081, hazard ratio = 10) and can predict the risk of resistance to carboplatin–paclitaxel combination for new patients (i.e., through leave-one-out cross-validation). We also confirm significant non-random predictive ability of our identified seven candidate pathways, when compared to seven pathways selected at random (random model *p*-value < 0.007) and show that our approach outperforms other commonly utilized methods (e.g., linear regression, support vector machine, and random forest) in identifying patients at risk of resistance to chemotherapy (Area Under the Receiver Operating Characteristics (AUROC) = 0.98)^24,29,30^. In addition, we demonstrate that our model is independent of, and is not affected by commonly used covariates (i.e., age, gender, and cancer stage at diagnosis) and by the known signatures of lung cancer aggressiveness (adjusted hazard ratio = 14, hazard *p*-value = 0.03). Finally, we extend our approach to additional chemo combinations (i.e., a combination of platinum-based cisplatin and plant alkaloid vinorelbine, and a combination of platinum-based oxaliplatin and antimetabolite agent fluorouracil) and additional cancer types (i.e., lung squamous cell carcinoma and colorectal adenocarcinoma)^4,31^ and demonstrate accuracy and general applicability of our approach (log-rank *p*-value < 0.03, hazard ratio > 3.5 across cancer types and chemotherapy-regimens). We propose that our model can be used to pre-screen patients and prioritize them for specific chemotherapy treatments.

## Results

### pathCHEMO overview

**Fig. 1:**
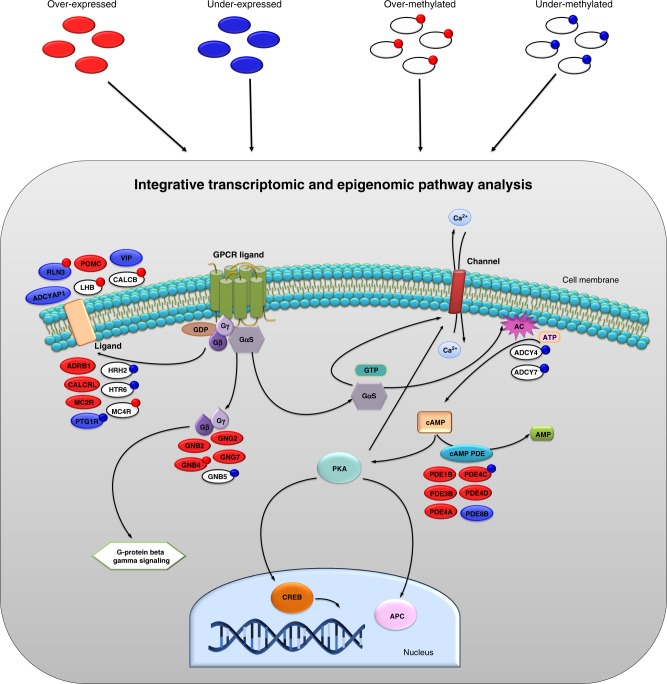
Schematic representation of pathway altered on both transcriptomic and epigenomic levels. Pathway genes affected on transcriptomic and epigenomic levels in G alpha (s) signalling events pathway are represented by ovals, where their colors correspond to either over-expression (red), under-expression (blue) or no differential expression (white). Small satellite circles represent over-methylation (red) or under-methylation (blue)

We have developed a genome-wide computational approach pathCHEMO that integrates mRNA expression and DNA methylation patient profiles to identify pathways altered on both transcriptomic and epigenomic levels (as demonstrated in Fig. 1) that differentiate poor from favorable response to chemotherapy-regimens. Here, we briefly outline the major steps of our integrative algorithm (also visualized in Supplementary Fig. 1). Step1: our algorithm identifies two groups of patients, which will be used to define a chemotherapy response signature: patients that failed a specific chemotherapy-regimens (e.g., developed metastasis within 1 year after therapy administration), and patients with favorable chemotherapy response (e.g., remained disease-free for more than 2 years after chemotherapy administration). Step 2: it compares transcriptomic (mRNA expression) and epigenomic (DNA methylation) profiles between these two groups of patients, which define differential transcriptomic signature and differential epigenomic signature of chemoresponse. Step 3: Such signatures are then individually subjected to signed and absolute valued pathway enrichment analyses, which are then integrated and define molecular pathways affected in either one direction (i.e., containing either over-expressed or under-expressed genes) or both directions (i.e., containing both over-expressed and under-expressed genes) enriched in the transcriptomic signature, and similarly pathways affected in either one direction or both directions on the epigenomic level, enriched in the epigenomic signature. Step 4: These transcriptomic and epigenomic pathway signatures are then integrated to define a set of pathways that control both transcriptomic and epigenomic programs disrupted in resistance. Step 5: Such candidate pathways and their read-out genes are subjected to validation studies, where they are evaluated for their ability to predict therapeutic response in independent patient cohorts, through multivariable survival analysis. Step 6: Finally, the identified pathways are used to assign individual risk of resistance for new incoming patients.

### Defining molecular signatures of chemoresponse

We tested our approach to evaluate response to standard-of-care doublet chemotherapy, which contains carboplatin and paclitaxel (i.e., carboplatin–paclitaxel), in LUAD patients. For this, we have analyzed clinical and molecular profiles of patient with LUAD in the TCGA clinical cohort^28^. To study primary resistance to this chemo combination, we specifically selected primary tumors from patients that did not receive any neoadjuvant therapy, were treated with adjuvant carboplatin–paclitaxel chemo regimen, and were further monitored for disease progression (*n* = 14) (Supplementary Table 1). Each patient that received carboplatin–paclitaxel was evaluated for his/her time to tumor relapse defined as time between the start of carboplatin–paclitaxel administration and a new tumor event (defined as tumor re-occurrence, local or distant metastases). To accurately uncover signal that differentiates poor from favorable treatment response, we employed an extreme-responder analysis, widely utilized by us^24,32,33^ and others^34,35^, where two groups of patients with drastically different treatment response (i.e., favorable response and poor response) are compared for differences in their molecular profiles to capture the most prominent molecular signal. To assure that the comparison groups are balanced with respect to initial age, gender, disease stage at diagnosis (i.e., initial disease aggressiveness), smoking status etc., we performed stratified sub-sampling (which identifies patient groups with similar distributions for these variables) and identified patients that experienced relapse within 1 year of carboplatin–paclitaxel start (i.e., poor response, *n* = 4); and patients that did not experience any events for more than 2 years (i.e., favorable response, *n* = 4) (Supplementary Table 2).

To uncover a complex interplay between transcriptomic and epigenomic mechanisms implicated in response to chemotherapy, we compared poor response and favorable response groups based on their mRNA expression and DNA methylation profiles using two-sample two-tailed Welch *t*-test^36^ and re-confirmed with fold change (see Methods), which defined carboplatin–paclitaxel response differential gene expression signature (Supplementary Data 1A) and carboplatin–paclitaxel response differential methylation signature (Supplementary Data 1B).

### Integrative analysis identified pathways of resistance

To understand molecular mechanisms that govern chemoresponse, we next sought to identify molecular pathways that control transcriptomic and epigenomic signatures of carboplatin–paclitaxel resistance (as in Fig. 1). For this, we subjected the carboplatin–paclitaxel response differential expression signature and carboplatin–paclitaxel response differential methylation signature to pathway enrichment analysis using the comprehensive C2 pathway database^37^ (which includes 833 pathways from REACTOME^38^, KEGG^39^, and BIOCARTA^40^ databases). Pathway enrichment was performed using Gene Set Enrichment Analysis (GSEA)^41^. This analysis estimated Normalized Enrichment Score (i.e., NES) for each of the 833 pathways, which reflects the extent to which each pathway is enriched in the treatment response signature, also referred to as pathway activity. A list of 833 pathways ranked by their enrichment (i.e., NESs) in the carboplatin–paclitaxel response differential expression signature defined carboplatin–paclitaxel response differential expression pathway signature and a list of 833 pathways ranked by their enrichment (i.e., NESs) in the carboplatin–paclitaxel response methylation signature defined carboplatin–paclitaxel response differential methylation pathway signature (see Methods). To account both for the pathways that have majority of their genes affected in the same direction (e.g., majority of genes being either over-expressed or under-expressed) and pathways that have genes affected in different directions: some genes affected in one direction (e.g., over-expressed) and some in an opposite direction (e.g., under-expressed), we have performed both signed and absolute valued pathway enrichment analysis with their subsequent integration (see Methods), which defined carboplatin–paclitaxel response composite expression pathway signature (Supplementary Data 2A) and carboplatin–paclitaxel response composite methylation pathway signature (Supplementary Data 2B).

**Fig. 2:**
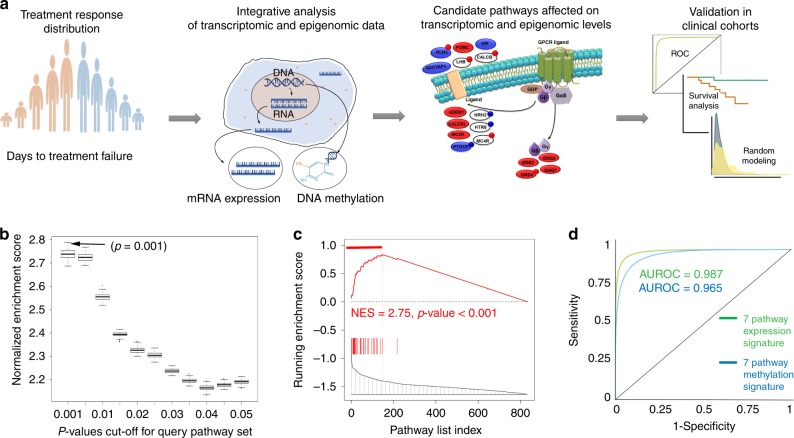
Integrative genome-wide transcriptomic and epigenomic analysis identifies candidate molecular pathways of chemotherapy response. **a** Schematic representation of the integrative transcriptomic and epigenomic analysis: first, patients are defined by their response to chemotherapy; second, our method integrates patients’ transcriptomic and epigenomic profiles; third, candidate pathways affected on both transcriptomic and epigenomic levels are identified; and finally, our method employs multi-modal validation of candidate pathways. **b** Box and whisker plot depicting *p*-value cutoff for query carboplatin–paclitaxel response composite methylation pathway signature (*x*-axis) and NESs from the corresponding GSEA comparison between composite methylation and expression pathways signatures (*y*-axis), based on analysis in TCGA-LUAD patient cohort. Arrow indicated optimal *p*-value threshold, which results in the strongest GSEA enrichment. **c** GSEA comparing carboplatin–paclitaxel response composite expression pathway signature (reference) and carboplatin–paclitaxel response composite methylation pathway signature (query, NES *p* ≤ 0.001), based on analysis in TCGA-LUAD patient cohort. Horizontal red bar indicates leading edge pathways altered on both transcriptomic and epigenomic levels. NES and *p*-value were estimated using 1000 pathway permutations. **d** ROC analysis comparing ability of the 7 candidate pathways to predict carboplatin–paclitaxel where their activity is defined based on their expression values (green) or methylation values (blue). AUROC is indicated

Further, to define interplay between complex mechanisms implicated in chemoresistance, we sought to identify molecular pathways that are affected on both transcriptomic (i.e., mRNA expression) and epigenomic (i.e., DNA methylation) levels and which would capture pathway genes affected: only on transcriptomic level, only on epigenomic level, or both levels (as in Fig. 1). To achieve this goal (Fig. 2a), we compared the carboplatin–paclitaxel response composite expression pathway signature (as a reference) and carboplatin–paclitaxel response composite methylation pathway signature (as a query pathway set) using GSEA (the threshold for the query pathway set at *p*-value ≤ 0.001 was selected as in Fig. 2b, see Methods), which identified seven molecular pathways with significant alterations on both transcriptomic and epigenomic levels (GSEA NES = 2.75, *p*-value < 0.001) (Fig. 2c, see Methods). These pathways included chemokine receptors bind chemokines, mRNA splicing, G alpha (s) signalling events, intestinal immune network for IgA production, metabolism of proteins, RNA degradation, and cell cycle mitotic.

To confirm that these identified seven molecular pathways are robust to the choice of the statistical methods used to define treatment response signatures, we have also performed our analysis using signatures defined using all DNA methylation sites and using non-parametric tests. First, we defined differential methylation signature with all DNA methylation sites considered (Supplementary Fig. 2a). Second, we defined differential methylation signature using fold change (Supplementary Fig. 2b). Finally, we defined both differential expression and differential methylation signatures using fold change (Supplementary Fig. 2c). Analyses using all of these signatures identified the same seven candidate pathways (GSEA NES > 2.45, *p*-value < 0.001), demonstrating robustness of our analysis regardless of the signature choice.

To investigate if mRNA expression or DNA methylation carries more weight in the predictive ability of our seven candidate pathways, we have performed Receiver Operating Characteristic (ROC) analysis^42^ based on pathway activities in each patient sample (i.e., through single-sample pathway analysis, see Methods), defined on either expression levels or methylation levels of the pathway genes (see Methods). The predictive ability was measured using Area under ROC (AUROC), which reflected how well each data type separates poor response and favorable response patients in the TCGA-LUAD patient cohort (the AUROC value of 0.5 indicates random predictor and 1 indicates a perfect predictor). Our analysis demonstrated that both expression levels (AUROC = 0.987) and methylation levels (AUROC = 0.965) of seven candidate pathways are highly predictive of poor response vs. favorable response separation (Fig. 2d), indicating that they both can be used to identify patients at risk of developing chemoresistance.

**Fig. 3:**
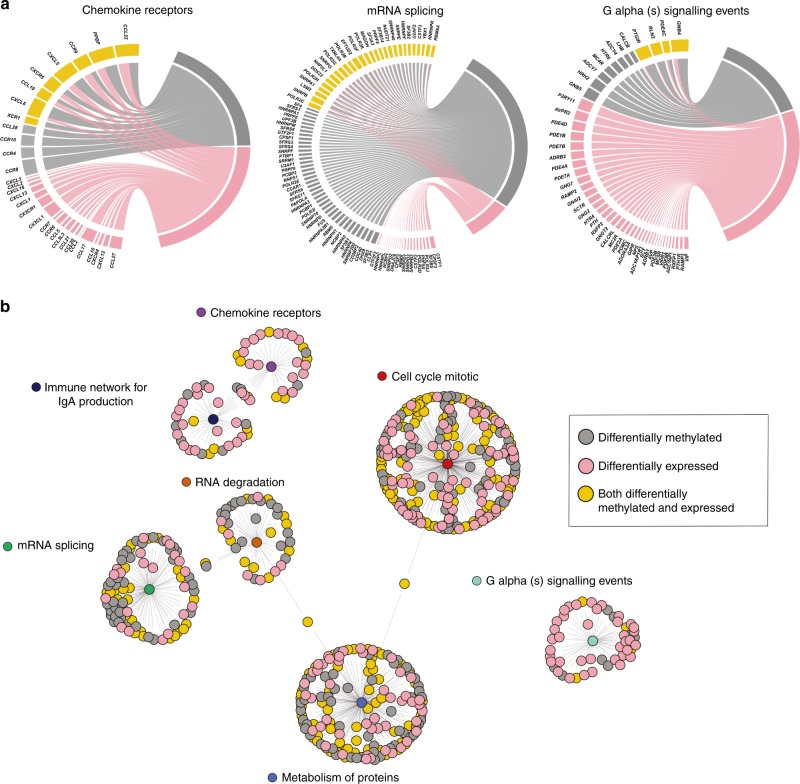
Transcriptomic and epigenomic alterations in candidate pathways of carboplatin–paclitaxel response. **a** Representative molecular pathways altered on both transcriptomic and epigenomic levels, visualized through circlize R package. Genes from the leading edge in each pathway are represented as differentially expressed (pink), methylated (gray) and both differentially expressed and methylated (yellow). Width of each connecting line is proportional to the extent of differential expression and differential methylation. Pathways are depicting as follows: chemokine receptors bind chemokines pathway (19 differentially expressed genes, four differentially methylated genes, and eight differentially expressed and methylated genes); mRNA splicing pathway (21 differentially expressed genes, 39 differentially methylated genes, and 28 differentially expressed and methylated genes); and G alpha (s) signaling events pathway (37 differentially expressed genes, eight differentially methylated genes, and four differentially expressed and methylated genes). **b** In the seven candidate pathway network representation, nodes correspond to the genes, which are connected to central pathway-membership circles (i.e., indicating pathway membership). Gene colors describe differential expression (pink), differential methylation (grey) and both differential expression and methylation (yellow). Network was constructed with ggnetwork R package

We further evaluated a topological structure of transcriptomic and epigenomic alterations within each identified pathway. Firstly, we examined to which extent genes from each pathway were affected on transcriptomic or on epigenomic levels (Fig. 3a, Supplementary Fig. 3, and Supplementary Data 2) and have observed that seven pathways exercised different patterns of transcriptomic and epigenomic alterations. For example, majority of genes from G alpha (s) signaling events pathway were altered on their mRNA level (i.e., Fig. 3a, nodes in pink) while genes from the mRNA splicing pathway were heavily altered on DNA methylation level (Fig. 3a, nodes in grey) and on both mRNA expression and DNA methylation levels (Fig. 3a, nodes in yellow). Secondly, we examined connectivity within and between the pathway genes, where an edge within the pathway corresponds to the pathway membership and connecting edge between pathways shows shared genes and demonstrated that our candidate pathways share little overlap (Fig. 3b). Finally, we examined differentially methylated sites harbored in genes from the seven pathways and evaluated their regions/locations on the genome (Supplementary Fig. 4a), where regions were defined as TSS200 (i.e., 200 base pairs upstream of transcription start site, TSS), TSS1500 (i.e., 1500 base pairs upstream of TSS200), 5’UTR, 1st exon, gene body, and 3’UTR. In fact, the majority of pathways have methylated sites overrepresented in TSS200 + TSS1500 regions, indicating a possible interaction with the transcription machinery binding at the promoter/enhancer regions^43^. An exception was Immune network for IgA production pathway, whose sites were heavily enriched in the gene body, indicating their potential interaction with alternative splicing machinery^44^ (Supplementary Fig. 4b).

### Validation in independent patient cohorts

**Fig. 4:**
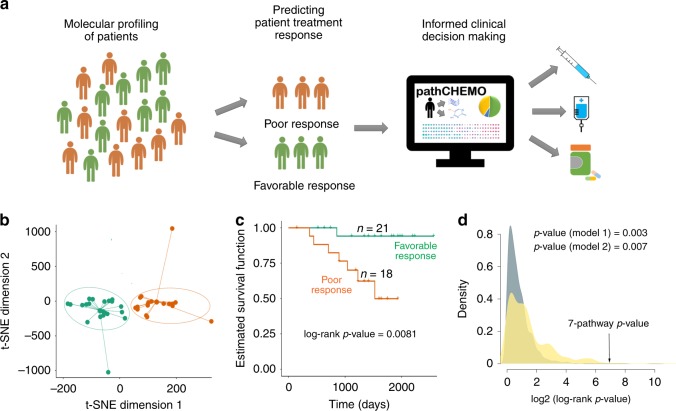
Candidate molecular pathways stratify patients based on response to carboplatin–taxane in an independent cohort. **a** Validation strategy, as follows: first, employment of molecular transcriptomic and epigenomic profiling of patients; second, predicting patients’ risk of developing chemoresistance; and finally, informed clinical decision making based on patients personalized risks. **b** t-SNE clustering of lung adenocarcinoma patients treated with carboplatin–taxane (e.g., paclitaxel) from the Tang et al. validation cohort (*n* = 39 biologically independent patient samples), based on activity levels of seven candidate pathways. Among two groups green group (*n* = 21 biologically independent patient samples) corresponds to patients with low composite activity levels of candidate pathways and orange group (*n* = 18 biologically independent patient samples) corresponds to patients with high composite activity levels of candidate pathways. **c** Kaplan–Meier survival analysis to estimate difference in response to carboplatin–taxane (e.g., paclitaxel) between two patient groups is identified in **b**. Log-rank *p*-value and number of patients in each group are indicated. **d** Two random models indicate non-random predictive ability of our model in the Tang et al. validation cohort: random model 1 (steel-blue) is defined based on to seven pathways selected at random, and random model 2 (goldenrod) is defined based on to equally sized patient groups selected at random

Our next essential step was to evaluate if the candidate molecular pathways can stratify patients based on the risk of failing chemotherapy in an independent, non-overlapping patient cohort (Fig. 4a). For this, we first considered a Tang et al. cohort^5^ (Supplementary Table 1) from the University of Texas MD Anderson Cancer Center, which contains LUAD primary tumor samples obtained at surgery (*n* = 39) collected between 1996 to 2007, followed by treatment with carboplatin and a taxane (e.g., paclitaxel) and monitored for further disease progression for 11 years. In this cohort, survival status during the clinical study (1996–2007) was used as a clinical endpoint and time to this event was calculated between the start of carboplatin–paclitaxel treatment to death (for patients with this event) or to follow-up (for censored patients). Similar to the analysis above, we evaluated activity levels of seven candidate pathways in each patient sample (i.e., through single-sample pathway analysis, see Methods) and employed t-Distributed Stochastic Neighbor Embedding (t-SNE) clustering^45^, which stratified patients into two groups based on pathway activity levels (Fig. 4b): one group with increased composite pathways’ activities (orange) and one group with decreased composite pathways’ activities (green). We then subjected these patient groups to Kaplan–Meier survival analysis and Cox proportional hazards model (Fig. 4c), which demonstrated that these groups had a significant difference in their response to carboplatin–paclitaxel (log-rank *p*-value = 0.0081, hazard ratio = 10) (see Methods).

To evaluate non-randomness of this result, we compared predictive ability of our candidate seven pathways to the predictive ability of seven pathways selected at random (see Methods), which demonstrated that ability of the candidate seven pathways to predict carboplatin–paclitaxel response is highly non-random compared to 10,000 randomly selected pathways (Fig. 4d, random model 1: *p*-value = 0.003). We paralleled this analysis with evaluation if patient groups stratified by our model are different in their treatment response compared to patient groups chosen at random, which were shown to be highly non-random (Fig. 4d, random model 2: *p*-value = 0.007).

Further, we simulated a situation when a new incoming patient is diagnosed with LUAD and needs to be assigned the risk of developing resistance to carboplatin–paclitaxel utilizing leave-one-out cross-validation (LOOCV)^46^ in the Tang et al. validation cohort^5^. In LOOCV, one patient is removed, and the model is trained on the rest of the patients. Then the patient that was removed is subjected to predictive analysis and is assigned a risk of developing resistance (i.e., simulating a scenario of a new incoming patient). This process is repeated for all patients (see Methods). LOOCV analysis demonstrated that our model has high accuracy in predicting poor and favorable carboplatin–paclitaxel response for a new incoming patient (Supplementary Fig. 5a).

Finally, to determine that our candidate pathways specifically distinguish carboplatin–paclitaxel response and not disease aggressiveness, we have evaluated if the pathways can also separate patients based on their lung cancer aggressiveness. For this, we evaluated the predictive ability of our candidate pathways on the LUAD patient cohorts that did not receive any treatment after surgery (we used these cohorts as negative controls). These datasets (Supplementary Table 1) included: Der et al.^47^ LUAD tumor samples (*n* = 127) collected through surgery between 1996 and 2005 at Princess Margaret Cancer Centre, and Tang et al.^5^ provisional cohort, which includes LUAD tumor samples (*n* = 94) collected through surgery between 1996 and 2007 at The University of Texas MD Anderson Cancer Center. These negative control patient cohorts did not receive any subsequent treatment but were monitored for disease progression (for Der et al. lung cancer-related death was used as a clinical endpoint and for Tang et al. survival status during the clinical study (1996–2007) was used as a clinical endpoint). Kaplan–Meier survival analysis on these datasets demonstrated that our candidate seven pathways did not separate patients based on the disease progression in both unstratified and stratified (i.e., based on tumor stages) analyses Der et al. (Supplementary Fig. 5b–d, log-rank *p*-value = 0.68), and Tang et al. (Supplementary Fig. 5e–g, log-rank *p*-value = 0.35) and are in fact specific for carboplatin–paclitaxel response.

### Comprehensive comparative analysis

To assess advantages of our approach, we have compared its predictive performance to other commonly utilized methods, including methods based on linear regression modeling, support vector machine (SVM), and random forest; and evaluated if our approach can be affected by commonly utilized covariates or known signatures of lung cancer aggressiveness.

**Fig. 5:**
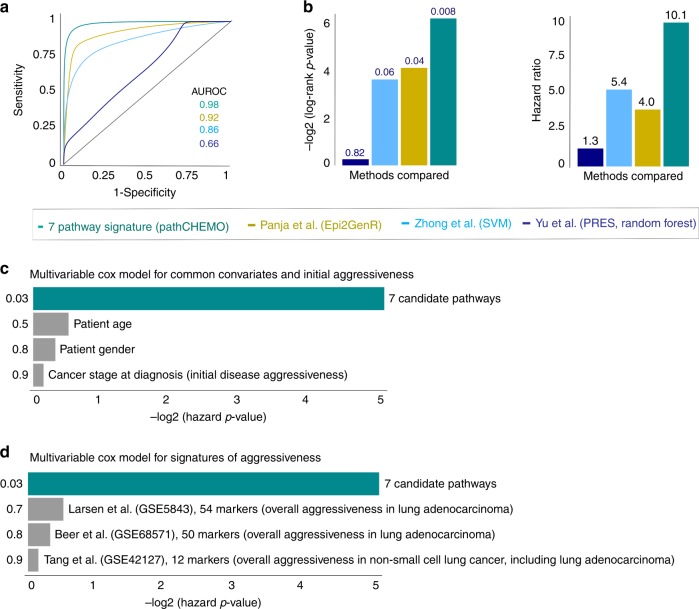
Comparative performance analysis confirms robust predictive ability of pathCHEMO. **a**, **b** Comparison of pathCHEMO (turquoise) to other commonly utilized methods, including Panja et al. Epi2GenR (yellow), Zhong et al. SVM (light blue), Yu et al. PRES random forest (dark blue) using (**a**) ROC analysis (with AUROC indicated) and (**b**) Kaplan–Meier and Cox proportional hazards model (with log-rank *p*-value and hazard ratio indicated) in Tang et al. validation cohort. **c** Multivariable Cox proportional hazards analysis demonstrating adjustment of seven candidate pathways for common covariates (i.e., age, gender and stage at diagnosis). Hazard *p*-value is indicated. **d** Multivariable Cox proportional hazards analysis demonstrating adjustment of seven candidate pathways for signatures of lung cancer aggressiveness, including Larsen et al. (54 lung adenocarcinoma markers), Beer et al. (50 lung adenocarcinoma markers), and Tang et al. (12 non-small cell lung cancer markers). Hazard *p*-value is indicated

First, to measure the advantage of our model over other commonly utilized methods, we have compared predictive performance of our model (see Methods) to Panja et al.^24^ method, Epi2GenR, based on linear regression integration between DNA methylation and mRNA expression patient profiles, which identified 35 site-gene pairs as candidate markers of carboplatin–paclitaxel response. Second, our model was compared to Zhong et al.^30^ method based support vector machine (SVM) analysis, which identified 104 candidate genes. Finally, our model was evaluated against Yu et al.^29^ method PRES, based on random forest algorithm, which identified three candidates of carboplatin–paclitaxel response. We first compared ability of the identified candidates from each method to separate patients with poor and favorable carboplatin–paclitaxel response in the Tang et al. dataset using ROC analysis, which demonstrated advantage of pathCHEMO over other commonly utilized methods (Fig. 5a, AUROC_pathCHEMO_ = 0.98, AUROC_Epi2GenR_ = 0.92, AUROC_SVM_ = 0.86, AUROC_PRES_ = 0.66). Furthermore, we compared ability of these methods to predict response to carboplatin–paclitaxel in the Tang et al. validation set (as above), through Kaplan–Meier survival analysis (Fig. 5b: log-rank *p*-value_pathCHEMO_ = 0.008, log-rank *p*-value_Epi2GenR_ = 0.04, log-rank p-value_SVM_ = 0.06, log-rank *p*-value_PRES_ = 0.82) and Cox proportional hazards model (Fig. 5b: hazard ratio_pathCHEMO_ = 10.1, hazard ratio_Epi2GenR_ = 4.0, hazard ratio_SVM_ = 5.4, hazard ratio_PRES_ = 1.3), which confirmed that pathCHEMO outperformed other commonly used methods in its ability to predict therapeutic response.

Second, to assure that our model is not affected by commonly utilized covariates (i.e., age, gender, and disease stage at diagnosis), we have evaluated their effect through multivariable (i.e., adjusted) Cox proportional hazards model^48^ on the Tang et al. dataset (see Methods), which demonstrated that these covariates are not predictive of treatment response and do not affect predictive ability of our model (Fig. 5c). Furthermore, to re-confirm this result we performed stratified Kaplan–Meier survival analysis; where we stratified the Tang et al. validation cohort into patient groups based on: age (< median age and ≥ median age); gender (i.e., female and male); and disease stage at diagnosis (stage I and stages II and III), which confirmed that ability of our model to predict chemotherapy response does not depend on commonly utilized covariates and is indeed indicative of a therapeutic response to carboplatin–paclitaxel (Supplementary Fig. 6).

Finally, to assure that our model is not affected by markers of overall tumor aggressiveness, we tested if any known prognostic signatures of lung cancer aggressiveness can predict carboplatin–paclitaxel response or affect predictive ability of our model. For this, we first selected known prognostic signatures of lung cancer aggressiveness including: Larsen et al.^49^ (54 prognostic markers); Beer et al.^50^ (50 prognostic markers); and Tang et al.^5^ (12 prognostic markers) (Fig. 5d) and utilized them in multivariable Cox proportional hazards model, as above. Our analysis demonstrated that these prognostic signatures were not predictive of carboplatin–paclitaxel response and did not affect the predictive ability of our seven candidate pathways (Fig. 5d).

### Pathway activity read-outs

Molecular pathways are comprised of multiple genes, which complicate their clinical applicability as markers of treatment response. To tackle this limitation, we looked for genes which could serve as read-outs of pathway’s activity implicated in therapeutic response. Specifically, we looked for genes inside each pathway, which were: first, altered on transcriptomic and/or epigenomic levels; second, correlated with pathway activity levels (i.e., NESs in each patient); and finally, associated with carboplatin–paclitaxel response (see Methods). This analysis identified seven read-out genes (i.e., *FGFR1OP*, *CCL22*, *CCR9*, *LSM7*, *PDE7A*, *CCT4*, and *POLR2C*), which: first, accurately reflected activity levels of their corresponding pathways; second, were associated with treatment response; and finally, achieved identical accuracy in predicting patients at risk of carboplatin–paclitaxel resistance (Supplementary Fig. 7, Supplementary Table 6). We propose that these seven read-out genes can be used as markers of carboplatin–paclitaxel response and can be easily adopted in the clinic.

### Model generalizability

**Fig. 6:**
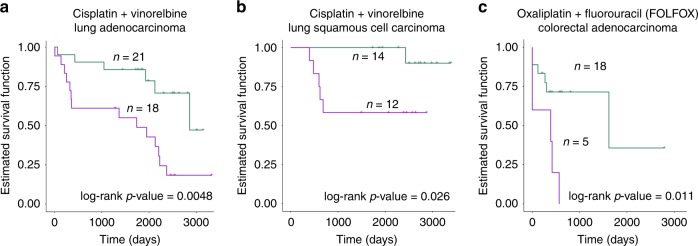
pathCHEMO accurately identifies pathways of treatment resistance across chemo-regimens and cancer types. Treatment related Kaplan–Meier survival analysis in **a** cisplatin–vinorelbine treated lung adenocarcinoma (LUAD) patients in the Zhu et al. patient cohort (*n* = 39 biologically independent patient samples), **b** cisplatin–vinorelbine treated lung squamous cell carcinoma (LUSC) patients in the Zhu et al. patient cohort (*n* = 26 biologically independent patient samples), and **c** FOLFOX (folinic acid, fluorouracil, and oxaliplatin) treated colorectal adenocarcinoma (COAD) patients in the Marisa et al. patient cohort (*n* = 23 biologically independent patient samples), demonstrating ability of identified candidate pathways (for each analysis) to predict treatment response. Log rank *p*-value and number of patients in each group are indicated

In order to test the general applicability of pathCHEMO, we applied our approach across additional chemotherapy combinations and cancer types. In particular, we extended pathCHEMO to: cisplatin–vinorelbine response in lung adenocarcinoma, cisplatin–vinorelbine response in lung squamous cell carcinoma, and folinic acid, fluorouracil, and oxaliplatin (i.e., FOLFOX) response in colorectal adenocarcinoma (Supplementary Table 3–5). First, we applied our approach to additional chemo-combination (i.e., cisplatin–vinorelbine) administered to lung adenocarcinoma (TCGA-LUAD) patients (Supplementary Table 3), which identified a set of three molecular pathways as markers of cisplatin–vinorelbine resistance (GSEA NES = 2.51, *p*-value < 0.001) (Supplementary Fig. 8a) and their corresponding read-out genes (Supplementary Table 6). These pathways included metabolism of nucleotides, actin Y, and ribosome pathways. We validated these predictions using the Zhu et al.^4^ cohort from the National Cancer Institute of Canada Clinical Trials Group (Supplementary Table 3), which contains LUAD tumor samples (*n* = 39) collected through surgery, for patients that received adjuvant cisplatin–vinorelbine, and demonstrated that three candidate pathways can predict poor and favorable cisplatin–vinorelbine response in patients with LUAD (lung cancer-related death used as a clinical endpoint) using Kaplan–Meier survival analysis and Cox proportional hazards model (Fig. 6a, log-rank *p*-value = 0.0048, hazard ratio = 3.64).

Next, we applied our approach to cisplatin–vinorelbine treated lung squamous cell carcinoma (TCGA-LUSC) patients (Supplementary Table 4) and identified a set of six molecular pathways (GSEA NES = 1.67, *p*-value < 0.001) (Supplementary Fig. 8b) including neuroactive ligand-receptor interaction, SLC-mediated transmembrane transport, transport of mature mRNA derived from an intron-containing transcript, cytokine-cytokine receptor interaction, DNA repair, and translation pathways and their corresponding read-out genes (Supplementary Table 6). We validated these predictions using the Zhu et al. patient cohort^4^ (Supplementary Table 4), which contains LUSC primary tumor samples (*n* = 26) collected through surgery, for patients that received adjuvant cisplatin–vinorelbine treatment, and demonstrated that six candidate pathways can accurately predict poor and favorable cisplatin–vinorelbine response in patients with LUSC (lung cancer-related death used as clinical endpoint) (Fig. 6b, log-rank *p*-value = 0.026, hazard ratio = 7.94).

Lastly, we applied our approach to patients with colorectal adenocarcinoma (TCGA-COAD) that received FOLFOX (i.e., folinic acid, fluorouracil, and oxaliplatin) combination (Supplementary Table 5), which identified five molecular pathways as markers of FOLFOX resistance (GSEA NES = 2.02, *p*-value < 0.001) (Supplementary Fig. 8c). These pathways included processing of capped intron containing pre mRNA, S phase, elongation and processing of capped transcripts, metabolism of proteins, and calcium signaling pathways and their corresponding read-out genes (Supplementary Table 6). We validated these predictions using an independent patient cohort, Marisa et al.^31^ (Supplementary Table 5) from the French National Cartes d’Identité des Tumeurs (CIT), which contains COAD tumor samples (*n* = 23) collected through surgery followed by adjuvant treatment with FOLFOX monitored for further disease progression (i.e., defined as locoregional or distant recurrence), and demonstrated that five candidate pathways can predict poor and favorable FOLFOX response in patients with COAD (Fig. 6c, log-rank *p*-value = 0.01, hazard ratio = 6.21). Interestingly, when evaluating overlaps between pathways across different chemo-treatments and cancers, we have noticed that even though some biological pathways might be overlapping (Supplementary Data 3), their overlapping genes exhibit totally different behaviors (e.g., are over-expressed for one chemo-regimen and are under-expressed for another etc.), thus demonstrating drastically different patterns of pathway dysregulations inherent for each specific chemo-regiment and for each cancer type.

Taken together, these analyses demonstrate the general applicability of our method across various chemotherapy-regimens and cancer types and builds a foundation for our long-term goal to enhance personalized therapeutic advice and improve patient care and clinical decision support at large.

## Discussion

We have introduced a systematic generalizable computational approach pathCHEMO to uncover molecular pathways that govern complex transcriptomic and epigenomic mechanisms implicated in chemotherapy response. Firstly, the distinguishing feature of pathCHEMO is in the identification of molecular pathways altered on both transcriptomic and epigenomic levels, which increases the likelihood of elucidating functionally relevant alterations. Secondly, the identified pathways constitute not only molecular markers for predictive analysis but also valuable candidates for therapeutic targeting to preclude or overcome resistance. Thirdly, our approach is generalizable and has been successfully applied to additional chemotherapy-regimens and cancer types, where it demonstrated the high accuracy of its predictions. Fourthly, pathCHEMO predicts patients at risk of developing resistance to specific chemotherapy, even prior to therapy administration, which builds a platform for optimal treatment planning and personalized therapeutic advice. Finally, to the best of our knowledge, pathCHEMO is the first computational predictive effort of its kind in chemotherapy resistance space, with near-term potential to improve informed clinical decision-making and cancer management.

We used pathCHEMO to elucidate mechanisms of resistance to carboplatin–paclitaxel chemotherapy in lung adenocarcinoma and identified seven molecular pathways implicated in resistance, including chemokine receptors bind chemokines, mRNA splicing, G alpha (s) signalling events, intestinal immune network for IgA production, metabolism of proteins, RNA degradation, and cell cycle mitotic pathways. Interestingly, paclitaxel resistance has been shown to be modulated by Hippo signaling pathway in breast cancer^51^, which is directly activated by our candidate G alpha (s) signalling events pathway^52^. Furthermore, chemokine receptors bind chemokines pathway is directly associated^53^ with cytokine and inflammatory response pathway, which modulates carboplatin resistance in ovarian cancer^54^. Finally, cell cycle mitotic pathway has been shown to be directly affected by paclitaxel^55^ and carboplatin–paclitaxel^56,57^ treatments in ovarian cancer. Thus, primary (i.e., before therapy administration) dysregulation in these pathways might affect drug mechanism of action and can be utilized to identify patients at risk of resistance.

Interestingly, one of the identified pathways, G alpha (s) signalling events pathway, is involved in mediation of extracellular signaling and activation of Protein Kinase A (PKA), a known player in cancer cell invasion and metastasis. Recently, PKA has been shown to play a central role in resistance to tamoxifen in breast cancer^58^, and disease progression in prostate cancer^59^. PKA has been known to contribute to lung cancer tumorigenesis by interacting with RAS oncogenic pathway and promoting epithelial-mesenchymal transition (EMT) during hypoxia. Several recent studies have confirmed the role of EMT as a key player in acquired (i.e., caused by the treatment) resistance to chemotherapy including acquired resistance to gemcitabine in pancreatic cancer^60^, to paclitaxel in ovarian cancer^61^, and to gefitinib in lung cancer^62^, emphasizing importance of further investigating EMT as a mechanism of primary resistance to chemotherapy in lung adenocarcinoma.

In addition to EMT, the development of neuroendocrine phenotype has been shown to be a major emerging player in acquired therapeutic resistance in lung cancer^63,64^. Recent studies have demonstrated that 50% of patients with metastatic lung adenocarcinoma, which were treated with erlotinib and acquired resistance to it, had a histological transformation to large cell neuroendocrine carcinoma (LCNEC), leading to increased metastatic burden and lethality^65,66^. Therefore, further investigation of the role of EMT and neuroendocrine markers and their interplay with transcriptomic and epigenomic molecular alterations are necessary for comprehensive understanding of complex mechanisms involved in resistance to chemotherapy and will contribute a central focus of our subsequent studies.

## Methods

### Lung adenocarcinoma patient cohorts

For this study, LUAD patient cohorts were obtained from publicly available data sources (Supplementary Table 1), which include. The Cancer Genome Atlas-Lung Adenocarcinoma (TCGA-LUAD)^28^, Tang et al. (GSE42127)^5^, Der et al. (GSE50081)^47^, and Zhu et al. (GSE14814)^4^ datasets. The primary LUAD patient cohort, utilized for reconstruction of transcriptomic and epigenomic signatures of chemoresistance, was obtained from The Cancer Genome Atlas (TCGA-LUAD) project^28^ and downloaded from the Genomics Data Commons database (GDC, https://portal.gdc.cancer.gov/) on February 2017. Clinical information (i.e., clinical file, follow-up, and treatment data) for these datasets were obtained from the TCGA GDC legacy archive (https://portal.gdc.cancer.gov/legacy-archive/).

### Gene expression and DNA methylation analysis

For RNA-seq analysis, we normalized and stabilized variance for raw RNA-seq counts using DESeq2 R package. DNA methylation values for each site were reported as *β* (Beta) values, which were subsequently converted to *M*-values as suggested in^67^ when parametric analysis was utilized, using beta2m function in Lumi R package.

### Defining signatures of chemotherapy response

To determine molecular characteristics that differ between poor response and favorable response, we defined signatures of treatment response on transcriptomic (i.e., differential expression) and epigenomic (i.e., differential methylation) levels between poor response and favorable response patient groups using two-sample two-tailed Welch *t*-test (t.test function in R) in R studio version 3.3.2, such that differential expression signature was defined as a list of genes ranked on their differential expression (i.e., *t*-test values) and differential methylation signature was defined as a list of genes based on the differential methylation of the corresponding site (i.e., *t*-test values). We coupled this analysis with signatures defined based on a fold change, and obtained similar results. For DNA methylation signature, we performed analysis two ways: selected one CpG site per gene through the coefficient of variation analysis, where a site with the highest coefficient of variation was selected for each gene; and considered all CpG sites for signature reconstruction, yielding similar results.

### Transcriptomic and epigenomic pathway enrichment analysis

To identify molecular pathways altered on transcriptomic and epigenomic levels (as in Fig. 1), we first performed pathway enrichment analysis on differential expression signature and differential methylation signature (as in Supplementary Fig. 1). For this, we used the comprehensive C2 pathway database^37^ (http://software.broadinstitute.org/gsea/msigdb), which includes 833 pathways from REACTOME^38^, KEGG^39^, and BIOCARTA^40^ databases, and implemented pathway enrichment analysis using Gene Set Enrichment Analysis (GSEA)^41^, where differential expression and differential methylation signatures were used as a reference and collection of genes from each pathway was used as a query gene set. Normalized Enrichment Scores (NESs), and p-values were estimated using 1,000 gene permutations. This analysis estimated NESs for each of the 833 pathways, which reflects the extent to which each pathway is enriched in the treatment response signature and defines a so-called pathway activity. Positive NES would reflect pathway enrichment in the over-expressed part of the signature (e.g., majority of pathway genes being over-expressed) and negative NES would reflect pathway enrichment in the under-expressed part of the signature (e.g., majority of pathway genes being under-expressed). We refer to such pathway enrichment analysis as signed as it considers over- and under-expression of genes (with direction). Signed pathway enrichment analysis was performed on the differential methylation signature of treatment response in the similar manner.

Further, to overcome limitations of such (i.e., signed) pathway enrichment analysis, which assumes that the pathway will be enriched only if majority of genes in the pathway are changed in the same direction (i.e., either over-expressed or under-expressed, but not both), we performed absolute valued analysis. For this, the pathway enrichment analysis was run on the absolute valued differential expression signature, where signature *t*-stat values are absolute valued to collapse positive and negative signature tails, as was previously done in^33^. In this case, positive NESs reflect enrichment in the differentially expressed part of the signature (which includes both over-expressed and under-expressed genes) and negative NESs reflect enrichment in the non-differentially expressed part of the signature (and are therefore not considered). This absolute valued pathway enrichment analysis discovers pathways whose genes might be changed in both directions (both over-expressed and under-expressed) as it estimates the enrichment in the differentially expressed tail of the signature (irrespective of sign). Such absolute valued pathway enrichment analysis defined NESs for each of 833 pathways, as above. Absolute valued pathway enrichment analysis was performed on the differential methylation signature of treatment response in the similar manner.

The next essential step was to then integrate NESs from signed and absolute valued pathway enrichment analysis so that for each pathway a final integrative NES was defined as an NES with the lowest *p*-value between signed and absolute valued pathway analyses (note, that negative NES values for absolute valued analysis are not considered as they reflect enrichment in the non-changed part of the signature). The advantage of such integration is two-fold: it captures pathways whose genes are strictly over-expressed or under-expressed in each pathway, and whose genes are changed in both directions (i.e., such pathway would contain genes that are over-expressed and genes that are under-expressed), thus increasing the probability to identify functionally relevant molecular determinants. Similar logic applies to the methylation signatures. Such integration of signed and absolute valued NESs defined composite expression pathway signature and composite methylation pathway signature.

### Transcriptomic and epigenomic pathway integration

We have employed GSEA to compare composite expression pathway signature and composite methylation pathway signature to identify pathways that are affected on both transcriptomic and epigenomic levels (i.e., belong to the leading edge from the GSEA analysis). To assure that we can identify pathways which are over-expressed and under-methylated; under-expressed and over-methylated; differentially expressed and differentially methylated etc., each pathway signature was ranked based on the absolute values of their NESs and used for subsequent GSEA comparative analysis.

For this pathway-based GSEA, we utilized composite expression pathway signature as a reference signature and top pathways from the composite methylation pathway signature as a query pathway set. To accurately define query pathway set, which should assure strongest enrichment between pathway signatures, we varied the threshold for the query pathway set between 0.001 and 0.05 (width of each step = 0.005) and estimated the strength of enrichment between the two signatures at each threshold. Since GSEA is a probabilistic algorithm, for each threshold, GSEA was run 100 times and average NES for the enrichment was reported. Threshold with the highest average NES then reflects the optimal threshold which corresponds to the strongest enrichment between the composite expression pathway signature and the composite methylation pathway signature, used for subsequent analysis. GSEA analysis between the composite expression pathway signature and the composite methylation pathway signature at the optimal threshold identified a set of pathways (e.g., for carboplatin–paclitaxel response LUAD, we identified 28 pathways) of treatment response altered on both transcriptomic and epigenomic levels.

One of the limitations of the pathways from the C2 collection is that they often represent a parent–child relationship, where a parent pathway (e.g., cell cycle) would encompass all genes in its child pathways (i.e., cell cycle phase). Such overlap produces data redundancy and can result in model overfitting as the same pathways are fit in the model repeatedly. To overcome this limitation and to eliminate pathways with heavy overlaps, we performed Fisher Exact Test (fisher.test function in R) and compared leading edge genes for each pair of pathways from our analysis (e.g., for all 28 pathways, resulting in (28 choose 2 = 378) comparisons). From each group of parent–children pathways which shared a large number of overlapping genes, we selected one representative pathway with the NES corresponding to the lowest *p*-value, which defined a final set of pathways (e.g., for carboplatin–paclitaxel response LUAD, we identified seven pathways) maximally non-overlapping non-redundant pathways used for subsequent analysis.

### Comparing expression and methylation predictive ability

To examine if, in our candidate pathways, both data types (i.e., mRNA expression or DNA methylation) have equivalent ability to predict therapeutic response, we compared the performance of candidate pathways utilizing their activity levels based on expression only and activity levels based on methylation only, separately. To compare pathway performances based on each data type, we first scaled both expression and methylation data matrices (i.e., *z*-scored on genes or sites) in the discovery (i.e., TCGA-LUAD) cohort, which defined single-sample differential expression and single-sample differential methylation signatures, respectively. Each sample was then used for signed and absolute valued pathway enrichment analysis (separately for expression and for methylation, as above), where each single-sample signature was used as a reference and genes from each of seven candidate pathways were used as a query set thus producing a pathway activity signature for each patient. These single-sample expression and methylation pathway signatures were then used to evaluate predictive ability of seven pathways (for expression and methylation, separately), using logistic regression modeling followed by ROC analysis. The logistic regression analysis was done using glm function and ROC analysis was done using pROC and ggplot2 packages in R.

### Validation and robustness in independent clinical cohorts

To evaluate clinical significance of the candidate molecular pathways, we examined their ability to predict patients at risk of chemoresistance in an independent clinical cohort from the Tang et al.^5^ dataset, and used survival status during the clinical study (1996–2007) as a clinical endpoint (time to event or follow-up was estimated between the start of carboplatin–paclitaxel treatment and death or follow-up, respectively; maximum time to event/follow-up is 2567 days). First, we estimated activity levels of the candidate pathways in the independent clinical Tang et al. cohort on a single-sample level, as above. The activity levels (i.e., NESs) of the candidate pathways were then subjected to t-Distributed Stochastic Neighbor Embedding (t-SNE) clustering^45^ (implemented through Rtsne package in R), a non-linear dimensionality reduction technique which chooses two similarity measures between pairs of points of high dimensional input space and low-dimensional embedding space. First, it constructs a probability distribution over the pairs of high dimensional space (i.e., seven-dimension in our case) in such a way that similar points are exhibited by nearby instances, while dissimilar points are exhibited by distant instances. Second, it constructs a similar probability distribution over the points in low-dimensional embedding space and tries to minimize the Kullback-Leibler divergence (i.e., KL divergence) between the high dimensional data and low-dimensional anticipated data at each point. Therefore, patients with similar pathway activity levels will be anticipated as nearby instances while patients with dissimilar pathway activity levels will be anticipated as dissimilar instances. The advantage of t-SNE lies in its ability to reduce dimensions from seven (maximum possible in our analysis) to two and effectively identify groups of patients that share similar pathway activity levels. This analysis stratified patients into two groups: a group with overall increased composite pathways’ activities and a group with overall decreased composite pathways’ activities. We then evaluated if these patient groups differ in their response to carboplatin–paclitaxel treatment using Kaplan–Meier survival analysis, and Cox proportional hazards model via survival, ggplot2 and survminer R packages.

In order to evaluate if a random set of pathways can perform as well as our identified seven pathways, we compared the predictive ability of our seven candidate pathways to the predictive ability of seven pathways selected at random. For this, we built a random model, where seven pathways were selected at random and their activity levels were utilized to stratify patients based on their treatment response, with subsequent evaluation using Kaplan–Meier survival analysis. Random selection was done 10,000 times and the empirical *p*-value was estimated as a number of times Kaplan–Meier log-rank *p*-value for seven candidate molecular pathways outperformed the results at random. We have also employed a second random model, where we evaluated the effect of selecting random patient groups.

Finally, to estimate the accuracy with which our model can predict treatment response for a new incoming patient, we simulated this process using leave-one-out cross-validation (LOOCV). In LOOCV, one patient is removed; and the model is trained on the rest of the patients. The patient that was removed is considered as a new incoming patient, subjected to predictive analysis, and is assigned a risk of developing resistance. This process was repeated for all patients. We implemented the predictive model for LOOCV using generalized linear modeling (e.g., utilizing multivariable logistic regression) through glm function and ggplot2 package in R.

### Comprehensive comparative analysis

To assess advantages of our approach, we have compared its predictive performance to other commonly utilized approaches, including linear regression modeling, support vector machine, and random forest; and evaluated if our approach can be affected by commonly used covariates or known signatures of lung cancer aggressiveness.

To demonstrate the advantages of our approach over other commonly utilized methods, we compared its performance: first, to Panja et al.^24^ method, Epigenomic and Genomic mechanisms of treatment Resistance (Epi2GenR), which utilized linear regression to integrate DNA methylation and mRNA expression data; second, to Zhong et al.^30^ method, based on support vector machine (SVM) algorithm which utilized mRNA expression patient profiles; and finally, to Yu et al.^29^ method, Personalized REgimen Selection (PRES) method, based on random forest machine learning approach which utilizes mRNA expression patient profiles. We followed the selection and cross-validation techniques suggested in each of the above publications to carefully compare their performance to our approach. Epi2GenR utilized the same signature as utilized in our study. To apply SVM and PRES correctly, we split our validation set into 70:30 proportion subsets, where 70% of the validation set were used for model training and 30% for model validation. Predictive ability of the identified candidates from each of these methods was evaluated using ROC, Kaplan–Meier survival, and hazard ratio analyses through survival, survcomp, and survminer packages in R.

Next, we evaluated if any of commonly used covariates (i.e., age, gender, and disease stage at diagnosis) and known signatures of lung cancer aggressiveness (from Larsen et al.^49^, Beer et al.^50^, and Tang et al.^5^ described above) can predict therapeutic response or can affect predictive ability of the identified seven candidate pathways. For this, we utilized the multivariable Cox proportional hazards model^48^ (using coxph function in R) and stratified Kaplan–Meier survival analysis through survival, and survminer packages in R.

### Pathway activity read-outs

To identify pathway read-outs, we looked for genes inside each pathway, which were altered on transcriptomic and/or epigenomic levels (i.e., belong to the leading edge from the pathway enrichment analysis); correlated with pathway activity levels (i.e., correlation between NESs and a candidate gene across all patients, measured by Pearson correlation, cor.test function in R); and associated with carboplatin–paclitaxel response (i.e., Cox proportional hazards model through coxph in R, using likelihood-ratio test as reliable for small sample sizes^68^). Likelihood-ratio test *p*-values were then combined with Pearson correlation p-values using Fisher’s method (metap R package) and utilized to make final gene selection. Visualization of the resulting read-outs was done using Cytoscape^69^.

### Model generalizability

To test the generalizability of our model, we applied our method to additional chemotherapy combinations (i.e., cisplatin–vinorelbine and oxaliplatin–fluorouracil) and additional cancer types (i.e., lung squamous cell carcinoma and colorectal adenocarcinoma) (Supplementary Table 3–5). In particular, we investigated response to: cisplatin (platinum-based alkylating chemotherapy) and vinorelbine (non-platinum-based plant alkaloid chemotherapy) response in lung adenocarcinoma (LUAD); cisplatin–vinorelbine response in lung squamous cell carcinoma (LUSC); and oxaliplatin (platinum-based alkylating chemotherapy), fluorouracil (antimetabolite chemotherapy) and, folinic acid (chemotherapy protective drug often given with fluorouracil to improves the binding; also known as leucovorin) (i.e., FOLFOX) response in colorectal adenocarcinoma (COAD).

For signature development, we utilized primary tumor samples from TCGA-LUAD/TCGA-LUSC/TCGA-COAD (*n* = 8), for patients without neo-adjuvant treatment (i.e., no pre-treatment), who received adjuvant chemotherapies of interest and were further monitored for new tumor events (as defined above).

For clinical validation of response to cisplatin–vinorelbine combination in LUAD we utilized the Zhu et al. patient cohort^4^ (GSE14814), which included LUAD tumors obtained at surgery (*n* = 39), treated with adjuvant cisplatin–vinorelbine chemotherapy. In this cohort, lung cancer-related death was used as a clinical endpoint and time to event was calculated between the start of cisplatin–vinorelbine treatment and lung-cancer-related death (for patients with this event) or to follow-up (for censored patients), with maximum time to event/follow-up 3390 days.

For clinical validation of response to cisplatin–vinorelbine combination in lung squamous cell carcinoma (LUSC) we utilized a different subset of patients from the Zhu et al. patient cohort^4^ (GSE14814), which were patient with LUSC whose tumors were obtained at surgery (*n* = 26) and who were treated with adjuvant cisplatin–vinorelbine chemotherapy. In this cohort, lung cancer-related death was used as a clinical endpoint and time to event was calculated between the start of cisplatin–vinorelbine treatment and lung-cancer-related death (for patients with this event) or to follow-up (for censored patients), with maximum time to event/follow-up 3318 days.

Finally, for validation of FOLFOX combination in colorectal adenocarcinoma (COAD) we utilized Marisa et al. patient cohort^31^ (GSE39582), which includes COAD tumors obtained at surgery (*n* = 23), treated with adjuvant FOLFOX chemotherapies. In this cohort, relapse-free survival (i.e., where relapse was defined as locoregional or distant recurrence) was used as a clinical endpoint and time to event was calculated between the start of FOLFOX treatment to relapse (for patients with this event) or to follow-up (for censored patients), with maximum time to event/follow-up 2790 days.

To investigate pathways overlaps, we employed Fisher Exact Test (fisher.test function in R) on the leading edge genes from the transcriptomic and epigenomic pathways (i.e., genes that contribute to the enrichment of biological pathways in corresponding signatures). All resulting p-values are corrected for multiple comparisons using FDR.

### Statistics and reproducibility

Statistical analyses and data visualization were conducted using R studio version 3.3.2. To define differential expression signatures, we utilized two-sample two-tailed Welch *t*-test^36^ for parametric estimates and fold change for non-parametric estimates. Signatures were compared using Gene Set Enrichment Analysis (GSEA)^41^, where NES and *p*-values were estimated using 1,000 gene/site/pathway permutations. Clustering of patients based on their pathway activity levels (i.e., NESs) was performed using t-Distributed Stochastic Neighbor Embedding (t-SNE) clustering^45^. To estimate the statistical difference in treatment response between patient groups, we used Kaplan–Meier survival analysis and Cox proportional hazards model. To estimate the association between gene and pathway activity levels, we employed Pearson correlation analysis. When needed, *p*-values were combined using Fisher’s method. Statistical significance of the overlap between sets was estimated using Fisher’s Exact Test. *P*-values were adjusted for multiple comparisons using FDR correction. To assure reproducibility of our results, we have deposited a SWEAVE executable document with necessary data objects for freely available download and analyses at http://license.rutgers.edu/technologies/2019-121_pathchemo^70^.

### Reporting summary

Further information on research design is available in the Nature Research Reporting Summary linked to this article.

## Supplementary information

Supplementary Information

Description of additional supplementary files

Supplementary Data 1

Supplementary Data 2

Supplementary Data 3

Reporting Summary

Peer Review File

## Data Availability

The data depicted in the main and supplementary figures are available in the Supplementary Information and Supplementary Data 1–3. All the data supporting the findings of this study were obtained from the following public repositories: The Cancer Genome Atlas (TCGA) downloaded from the Genomics Data Commons data portal (GDC, https://portal.gdc.cancer.gov/); clinical information pertaining TCGA dataset downloaded from the TCGA GDC legacy archive (https://portal.gdc.cancer.gov/legacy-archive/); and all other datasets used for our analyses downloaded from the Gene Expression Omnibus (GEO): Tang et al. (GSE42127), Der et al. (GSE50081), Zhu et al. (GSE14814), Marisa et al. (GSE39582). C2 pathway database can be freely downloaded from MSigDB (http://software.broadinstitute.org/gsea/msigdb).

## Code availability

The code and data objects are combined into SWEAVE executable document for user-friendly download and result reproducibility, which can be freely accessed and downloaded from http://license.rutgers.edu/technologies/2019-121_pathchemo^70^.
