# Supplementary material for: pathCHEMO, a generalizable computational framework uncovers molecular pathways of chemoresistance in lung adenocarcinoma
Source: Commun Biol. 2019 Sep 6;2:334. doi: 10.1038/s42003-019-0572-6 (PMC6731276; doi:10.1038/s42003-019-0572-6)
Supplement: Supplementary file 1 — Supplementary Information [file 42003_2019_572_MOESM1_ESM.pdf]

pathCHEMO, a generalizable computational framework uncovers molecular pathways of chemoresistance in lung adenocarcinoma

Epsi *et al.*

Supplementary Figures

Supplementary Figure 1. Schematic flow representation of pathCHEMO.

Supplementary Figure 2, related to Fig 2. Comparative testing of treatment response signatures demonstrates their robustness.

Supplementary Figure 3, related to Fig 3. Transcriptomic and epigenomic alterations in selected candidate molecular pathways of carboplatin-paclitaxel resistance.

Supplementary Figure 4, related to Fig 3. Region-based analysis of differentially methylated sites in 7 candidate pathways.

Supplementary Figure 5, related to Fig 4. Candidate molecular pathways predict response to carboplatin-taxane and are not predictive of lung cancer aggressiveness.

Supplementary Figure 6, related to Fig 5. Stratified Kaplan-Meier survival analysis demonstrates independence of the candidate pathways from the common covariates.

Supplementary Figure 7, related to Fig 3. Network representation of candidate molecular pathways with their read-out genes.

Supplementary Figure 8, related to Fig 6. Identification of pathways of treatment resistance across chemo-regimens and cancer types.

Supplementary Tables

Supplementary Table 1. Clinical and pathological features of lung adenocarcinoma patient cohorts treated with carboplatin-paclitaxel, used for discovery, validation, and negative controls.

Supplementary Table 2. Clinical profiles of carboplatin-paclitaxel treated patients with poor (n = 4) and favorable (n = 4) treatment response from the TCGA-LUAD cohort.

Supplementary Table 3. Clinical and pathological features of lung adenocarcinoma patient cohorts treated with cisplatin-vinorelbine, used for discovery and validation.

Supplementary Table 4. Clinical and pathological features of lung squamous cell carcinoma patient cohorts treated with cisplatin-vinorelbine, used for discovery and validation.

Supplementary Table 5. Clinical and pathological features of colorectal adenocarcinoma patient cohorts treated with FOLFOX (folinic acid, fluorouracil, oxaliplatin), used for discovery and validation

Supplementary Table 6. Identified candidate pathways (carboplatin-paclitaxel treated LUAD, cisplatin-vinorelbine treated LUAD, cisplatin-vinorelbine treated LUSC, and FOLFOX (folinic acid, fluorouracil, oxaliplatin) treated COAD) readout, and contribution to cancer.

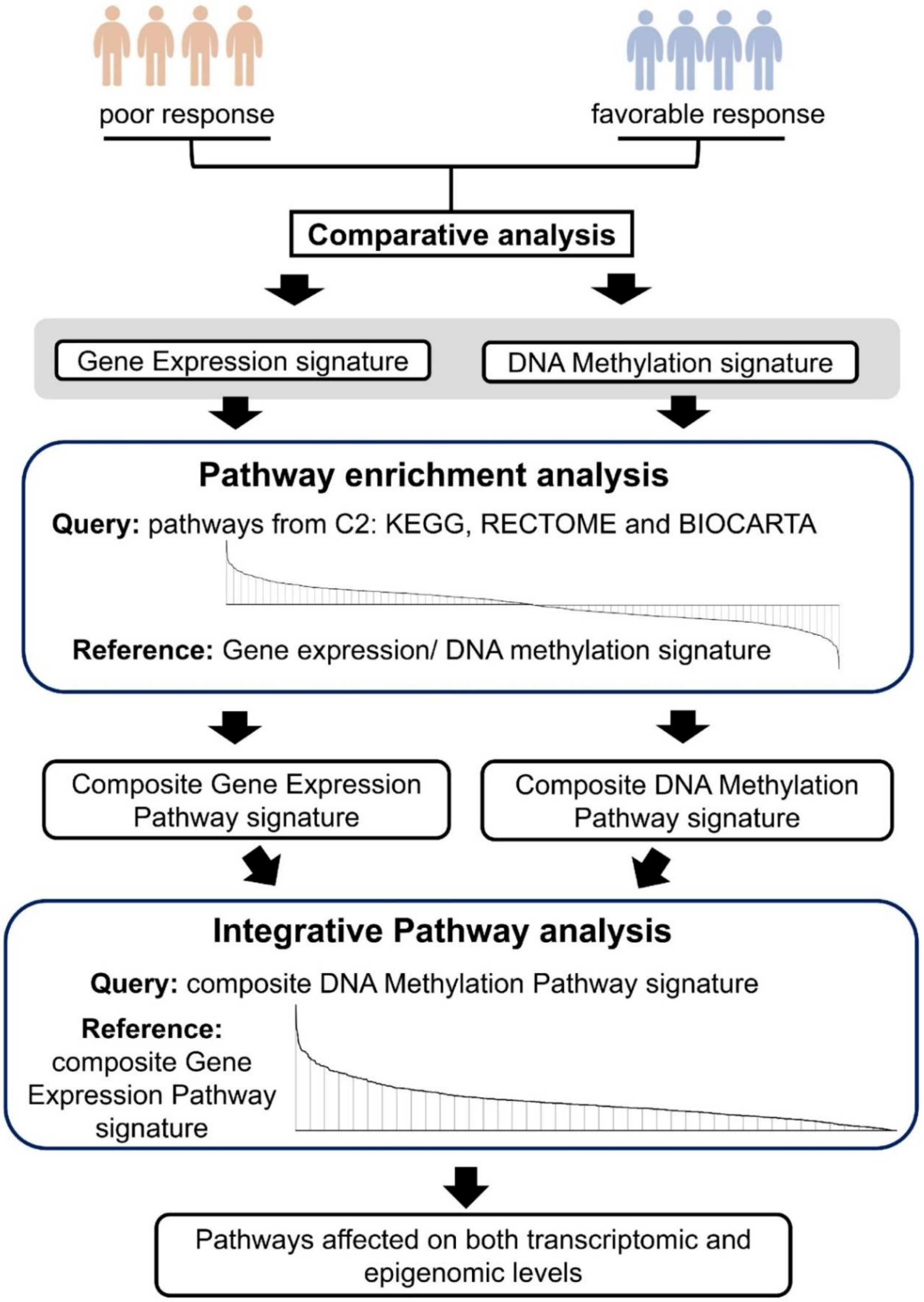
poor response
favorable response
Comparative analysis
Gene Expression signature
DNA Methylation signature
Pathway enrichment analysis
Query: pathways from C2: KEGG, RECTOME and BIOCARTA
Reference: Gene expression/ DNA methylation signature
Composite Gene Expression Pathway signature
Composite DNA Methylation Pathway signature
Integrative Pathway analysis
Query: composite DNA Methylation Pathway signature
Reference: composite Gene Expression Pathway signature
Pathways affected on both transcriptomic and epigenomic levels

Supplementary Figure 1 Schematic flow representation of pathCHEMO. Schematic flow of pathCHEMO approach.

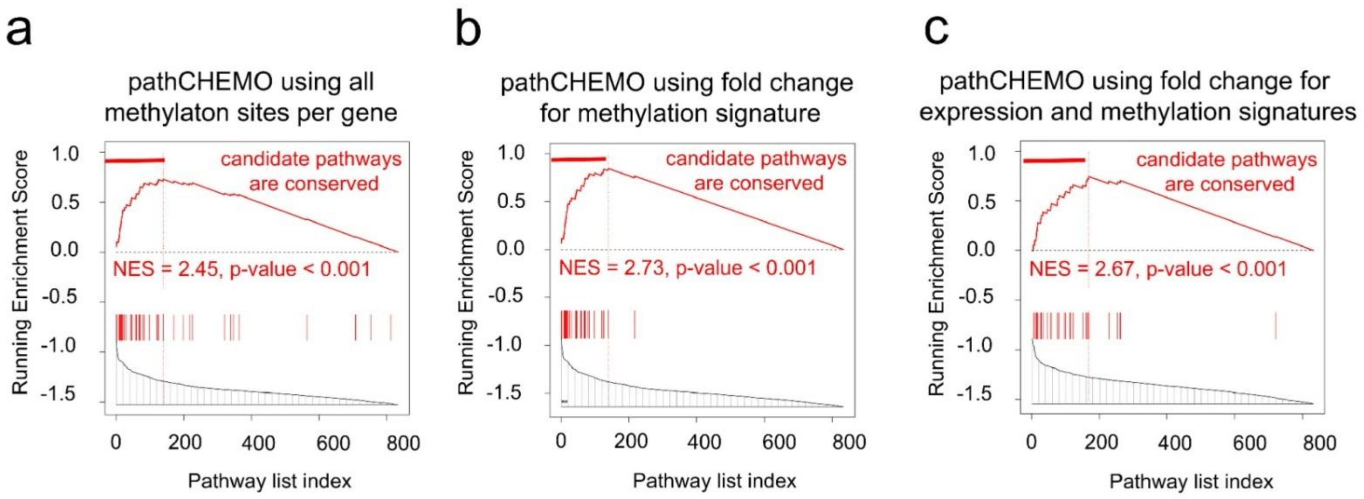

Supplementary Figure 2, related to Fig. 2 Comparative testing of treatment response signatures demonstrates their robustness. GSEAs comparing **(a)** treatment response composite expression pathway signature (reference) and treatment response composite methylation pathway signature constructed considering all CpG DNA methylation sites (query), **(b)** treatment response composite expression pathway signature (reference) and treatment response composite methylation pathway signature(query), where methylation signature was defined using fold change, and **(c)** treatment response composite expression pathway signature (reference) and treatment response composite methylation pathway signature (query), where both signatures were defined using fold change. Horizontal red bars indicate leading edge pathways altered on both transcriptomic and epigenomic levels. NES and p-value were estimated using 1,000 pathway permutations.

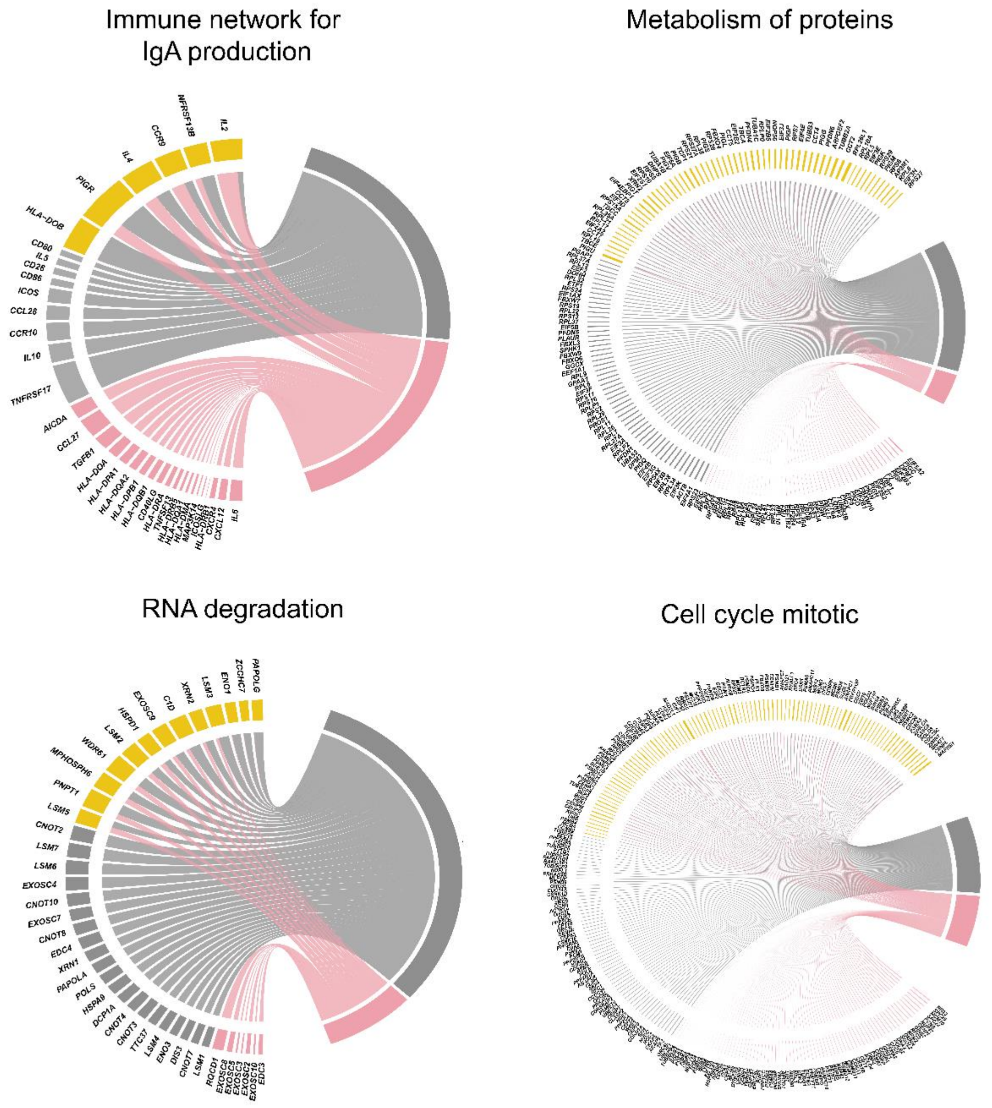

Supplementary Figure 3, related to Fig. 3 Transcriptomic and epigenomic alterations in selected candidate molecular pathways of carboplatin-paclitaxel resistance. Representative molecular pathways altered on both transcriptomic and epigenomic levels. Genes from the leading edge in each pathway are represented as differentially expressed (pink), methylated (grey) and both differentially expressed and methylated (yellow). Width of each connecting line is proportional to the extent of differential expression and differential methylation. Pathways are depicting as follows: (i) intestinal immune network for IgA production pathway (20 differentially expressed genes, 9 differentially methylated genes, and 6 differentially expressed and methylated genes), (ii) metabolism of proteins pathway (47 differentially expressed genes, 53 differentially methylated genes, and 62 differentially expressed and methylated genes), (iii) RNA degradation pathway (7 differentially expressed genes, 21 differentially methylated genes, and 13 differentially expressed and methylated genes), and (iv) cell cycle mitotic pathway (75 differentially expressed genes, 64 differentially methylated genes, and 100 differentially expressed and methylated genes). Pathways were visualized using circlize[1] package in R.

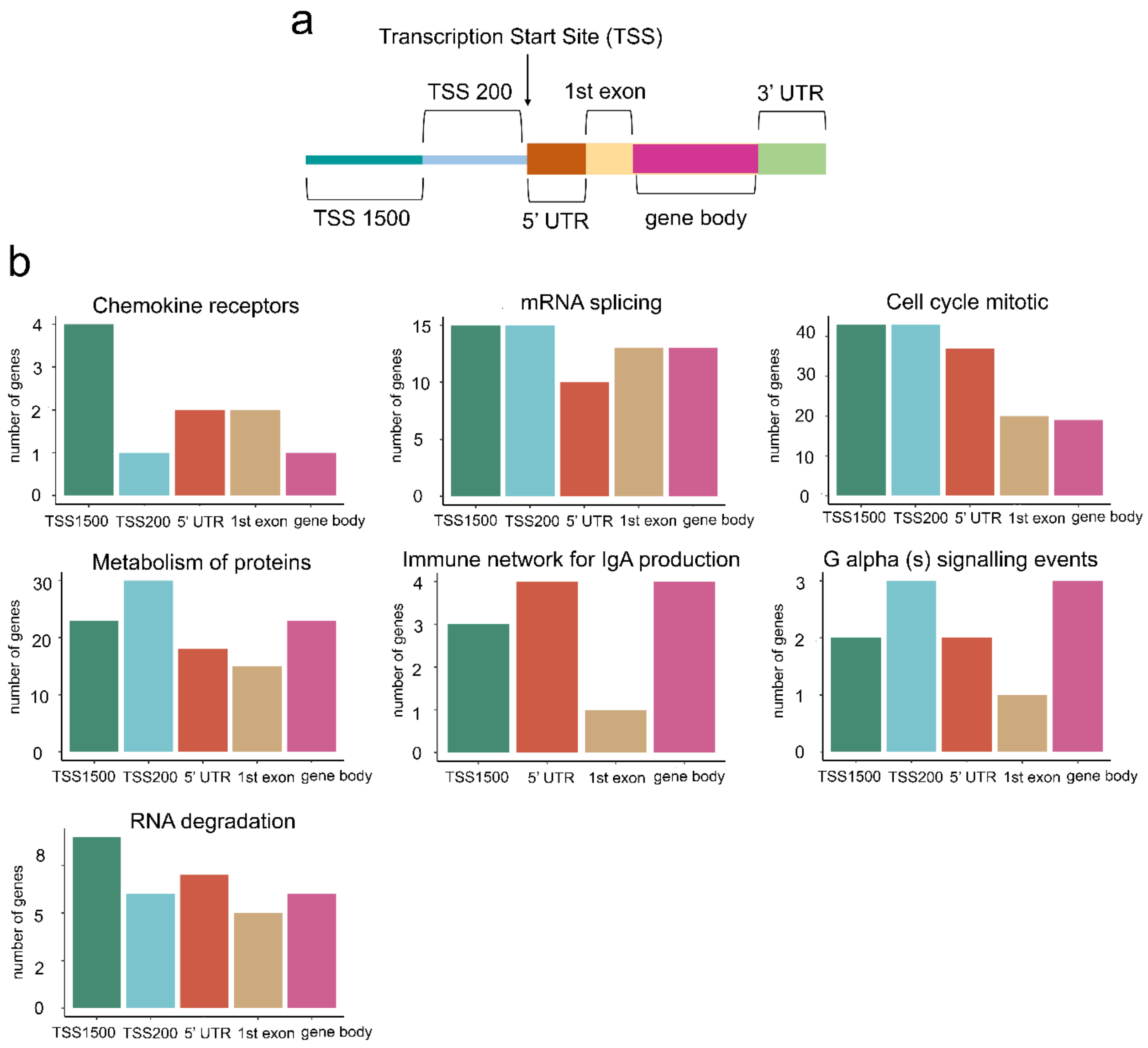

Supplementary Figure 4, related to Fig. 3 Region-based analysis of differentially methylated sites in 7 candidate pathways. (a) Schematic representation of regions (TSS1500, TSS200, 5'UTR, first exon, gene body, and 3'UTR) used to profile differentially methylated sites in the HumanMethylation450 array. **(b)** Bar plot representation of region distribution for pathway genes harboring differentially methylated sites.

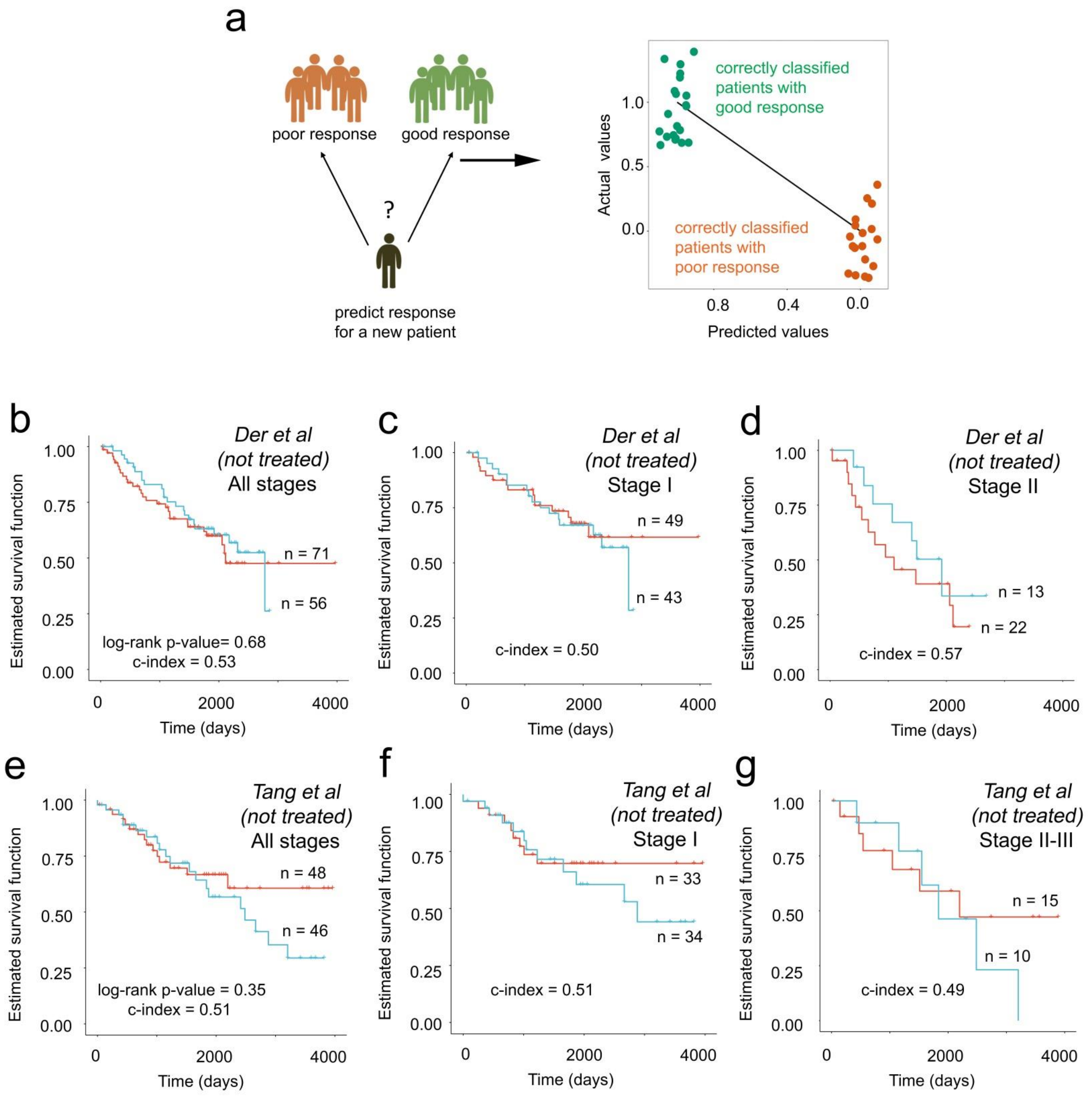

Supplementary Figure 5, related to Fig. 4 Candidate molecular pathways predict response to carboplatin-taxane and are not predictive of lung cancer aggressiveness. (a) Leave-one-out cross-validation (LOOCV) in the Tang et al. (n = 39) validation cohort. Correctly predicted patients with favorable response to carboplatin-taxane (e.g., paclitaxel) (green) and patients with poor response to carboplatin-taxane (e.g., paclitaxel) (orange) are indicated. **(b-g)** Kaplan-Meier survival analysis shows no significant difference between untreated patients based on the overall lung cancer aggressiveness in **(b-d)** Der et al. (n = 127) and **(e-g)** Tang et al. (n = 94) observational (i.e., not treated) patient cohorts. Log-rank p-value and the number of patients in each group are indicated.

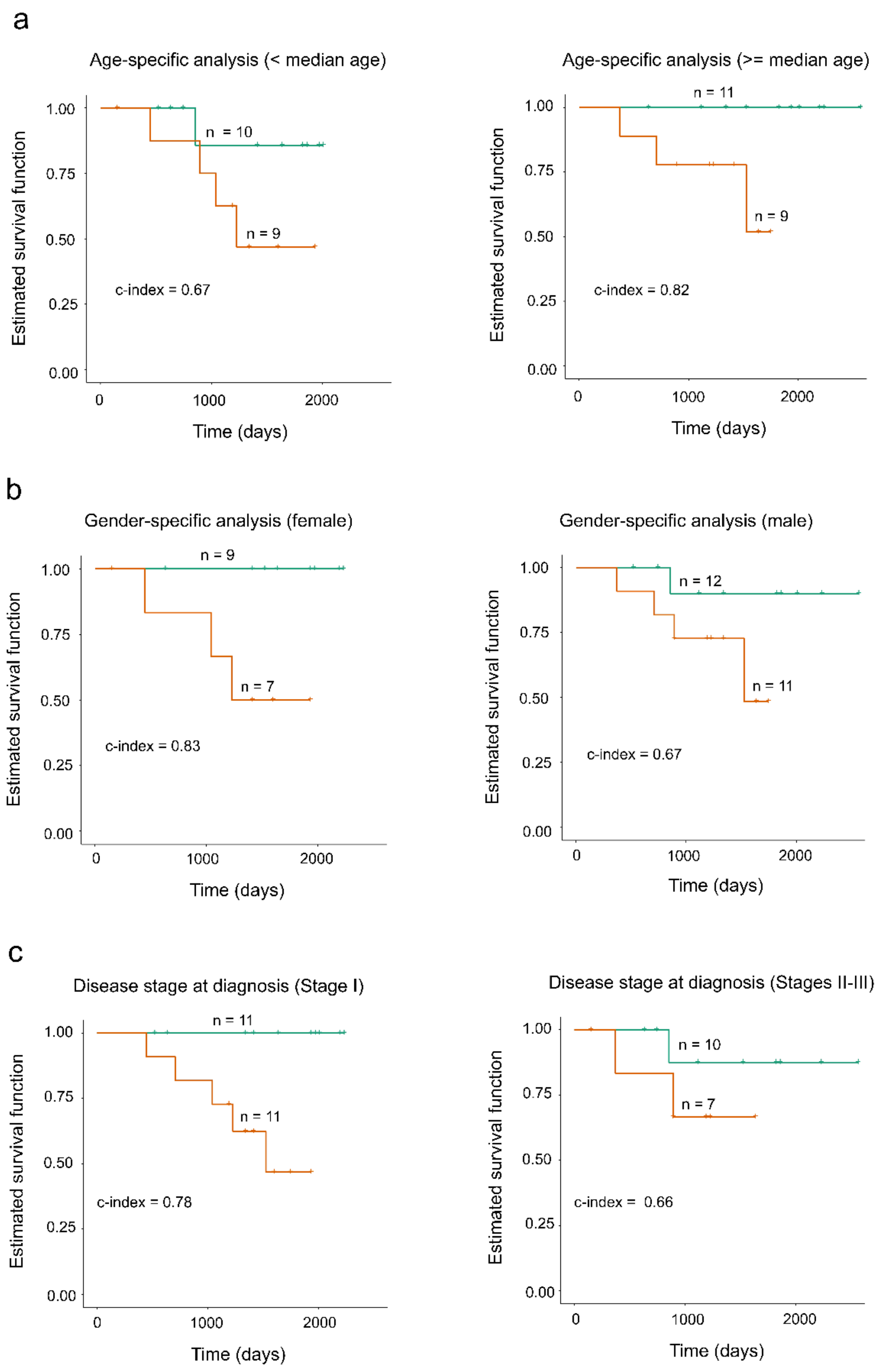

Supplementary Figure 6, related to Fig. 5 Stratified Kaplan-Meier survival analysis demonstrates independence of the candidate pathways from the common covariates. Stratified Kaplan-Meier survival analysis in the Tang et al. patient cohort (n = 39) based on common prognostic covariates: **(a)** age-specific analysis (greater than and less than median age); **(b)** gender-specific analysis (female and male), and **(c)** diseases stage at diagnosis (I and II-III). C-index and number of patients in each group are indicated.

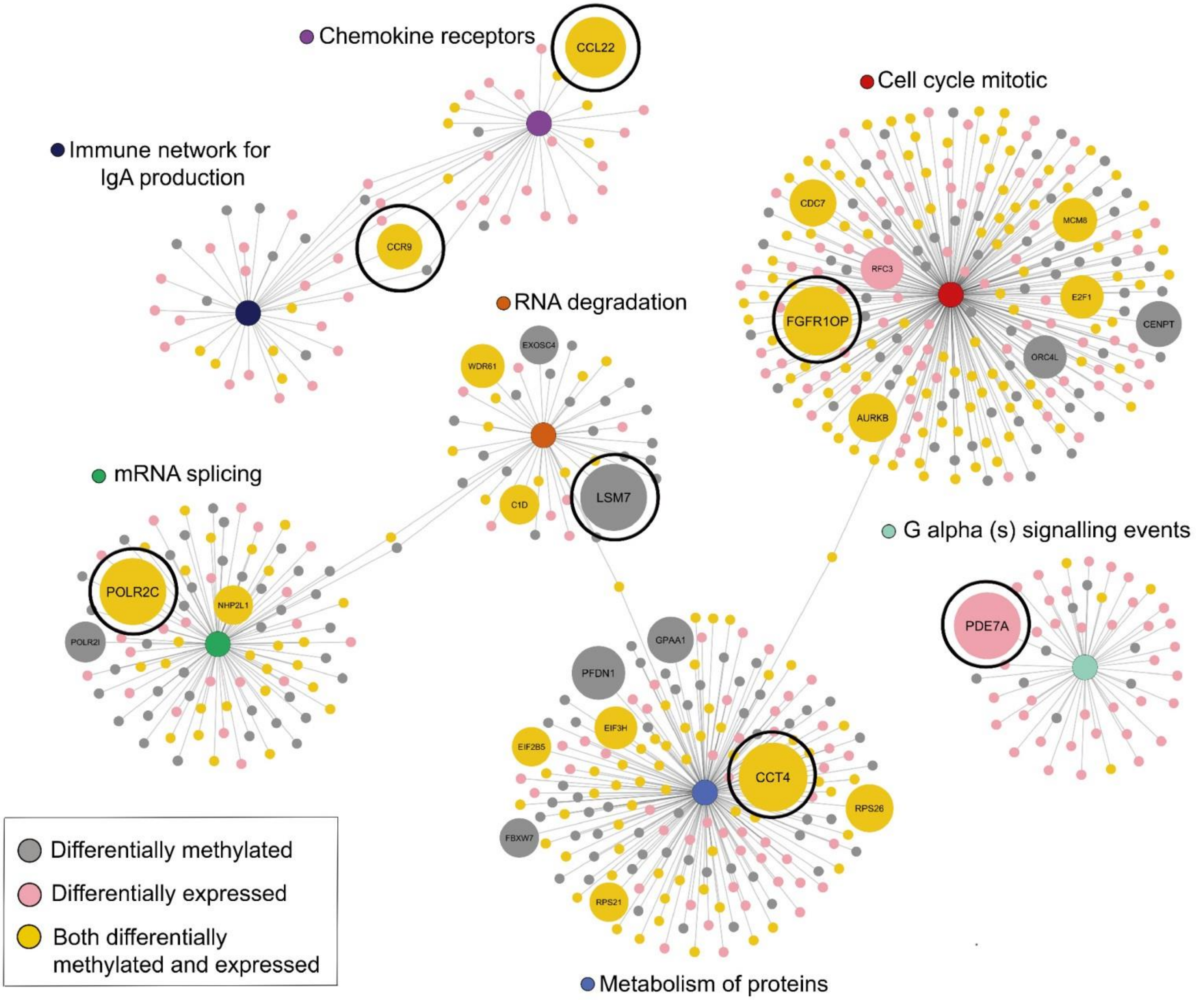

Supplementary Figure 7, related to Fig. 3 Network representation of candidate molecular pathways with their read-out genes. Network representation of the candidate pathways, where leading edge genes correspond to nodes and their sizes indicates –log based 2 of Fisher's combined p-values (i.e., combining likelihood-ratio test p-value for association with treatment response and Pearson correlation p-value for correlation with pathway activity). Largest nodes correspond to readout genes, for each pathway. Gene colors depict differential expression (pink), differential methylation (grey), and both differential expression and methylation (yellow).

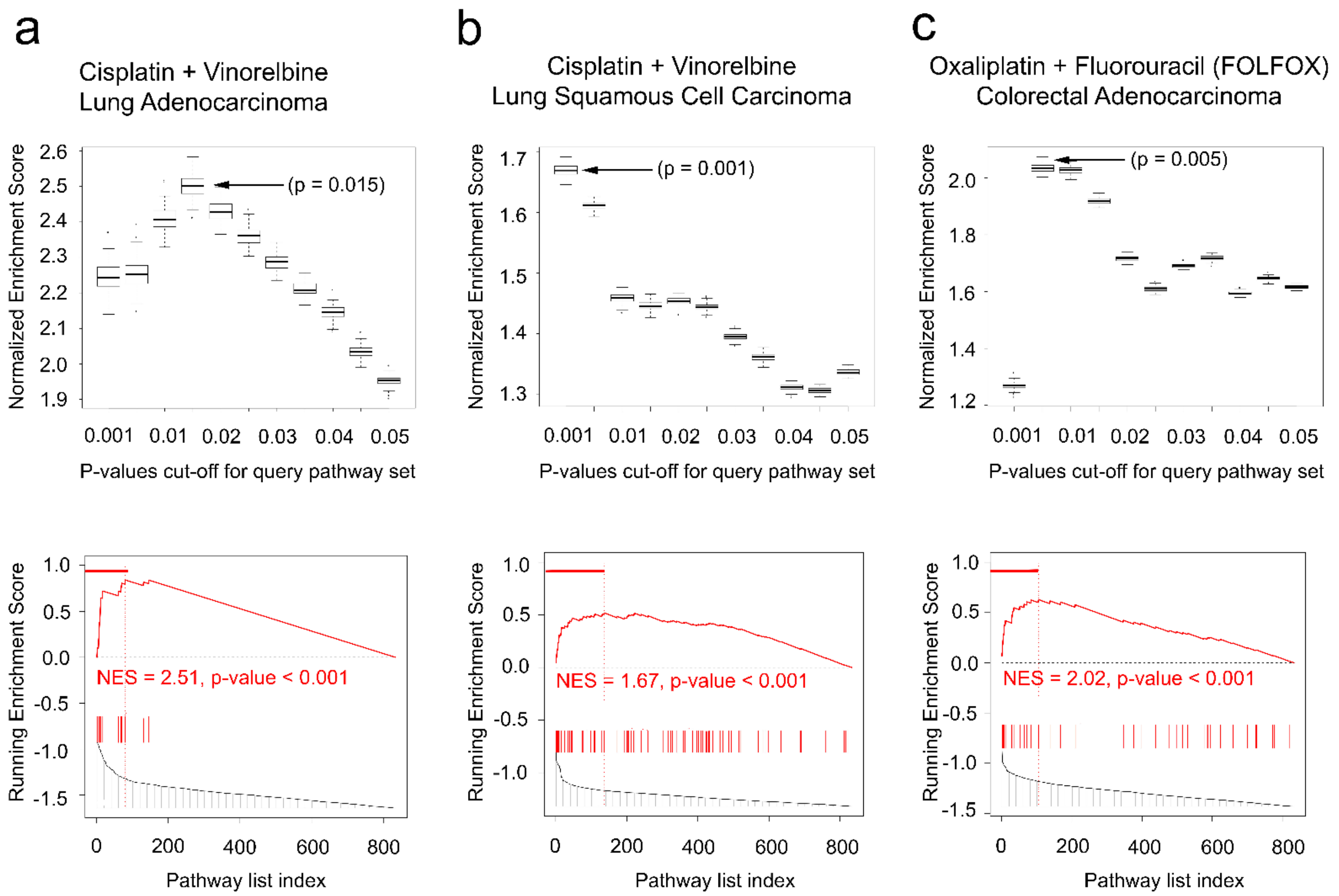

Supplementary Figure 8, related to Fig. 6 Identification of pathways of treatment resistance across chemo-regimens and cancer types. pathCHEMO discovery in **(a)** cisplatin-vinorelbine treated lung adenocarcinoma patients (TCGA-LUAD), **(b)** cisplatin-vinorelbine treated lung squamous cell carcinoma patients (TCGA-LUSC), and **(c)** FOLFOX (folinic acid, fluorouracil, and oxaliplatin) treated colorectal adenocarcinoma patients (TCGA-COAD). (i-iii) Box and whisker plots depicting p-value cutoff discovery for query treatment response composite methylation pathway signature (x-axis) and NESs from the corresponding GSEA comparison between treatment response composite methylation and expression pathways signatures (y-axis). Arrows indicate optimal p-value thresholds, resulting in the most significant GSEA enrichment. (iv-vi) GSEAs comparing indicated treatment response composite expression pathway signatures (reference) and indicated treatment response composite methylation pathway signatures (query). Horizontal red bars indicate leading edge pathways altered on both transcriptomic and epigenomic levels. NES and p-value were estimated using 1,000 pathway permutations.

Supplementary Table 1. Related to Fig. 2-5, Supplementary Figures 2-7. Clinical and pathological features of lung adenocarcinoma patient cohorts treated with carboplatin-paclitaxel, used for discovery, validation, and negative controls.

	**Signature discovery**	**Validation**	**Negative controls**	
**Description**	TCGA	Tang et al. (treated)	Tang et al. (not treated)	Der et al. (not treated)
**Accession #**	TCGA-LUAD[2]	GSE42127[3]	GSE42127[3]	GSE50081[4]
**Platform**	Illumina HiSeq 2000 (mRNA expression)<br>Illumina Infinium Human Methylation (HM450) array (DNA methylation)	Illumina HumanWG-6 v3.0 expression beadchip	Illumina HumanWG-6 v3.0 expression beadchip	Affymetrix Human Genome U133 Plus 2.0 Array
**Patients**	14	39	94	127
**Sample collection**	surgery	surgery	surgery	surgery
**Histological subtype**				
mixed	1	NA	NA	NA
acinar	1	NA	NA	NA
NOS	12	NA	NA	NA
**Anatomic Site**				
Left-Upper	5	NA	NA	NA
Left-Lower	2	NA	NA	NA
Right-Lower	1	NA	NA	NA
Right-Middle	2	NA	NA	NA
Right-Upper	4	NA	NA	NA
**Gender**				
Female	9	16	49	62
Male	5	23	45	65
**Tumor Stage (Pathological)**				
IA	NA	1	31	36
IB	1	21	36	56
II	NA	NA	NA	NA
IIA	1	1	5	7
IIB	4	5	11	28
IIIA	4	3	4	NA
IIIB	1	8	5	NA
IV	1	NA	1	NA
NA	2	NA	1	NA
**Smoking Status**				
1	2	NA	NA	NA
2	4	NA	NA	NA
3	3	NA	NA	NA
4	5	NA	NA	NA

Notes: NA = Not available, NOS = Not otherwise specified.
Smoking status: 1 = lifelong non-smoker (<100 cigarettes smoked in Lifetime), 2 = current smoker (includes daily smokers and non-daily smokers (or occasional smokers), 3 = current reformed smoker for > 15 years, 4 = current reformed smoker for ≤ 15 years.

Supplementary Table 2, related to Fig. 2-3 and Supplementary Figures 2-4, 7. Clinical profiles of carboplatin-paclitaxel treated patients with poor (n = 4) and favorable (n = 4) treatment response from the TCGA-LUAD cohort.

Treatment response	Patient ID	Time to event or follow-up (days)	Age	Gender	Disease stage at diagnosis	Smoking status	# pack years	Observed treatment related event or follow-up
poor response	6712	116	71	male	IIA	4	NA	new tumor event
	5051	122	42	female	IIIA	4	30	new tumor event
	6979	138	59	female	IIB	3	NA	new tumor event
	A4VP	153	66	female	IIIA	4	20	new tumor event
favorable response	4666	744	52	female	IV	4	10	no event, follow-up
	5899	784	58	male	IIA	2	NA	no event, follow-up
	1678	1,120	70	female	IIB	3	20	no event, follow-up
	1596	2,031	55	male	IIB	2	50	no event, follow-up

Notes: NA = not available.
Smoking status: 1 = lifelong non-smoker (< 100 cigarettes smoked in Lifetime), 2 = current smoker (includes daily smokers and non-daily smokers (or occasional smokers), 3 = current reformed smoker for > 15 years, 4 = current reformed smoker for ≤ 15 years, 5 = current reformed smoker, duration not specified, and 6 = smoking history not documented.

Supplementary Table 3, related to Fig. 6, Supplementary Figure 8 Clinical and pathological features of lung adenocarcinoma patient cohorts treated with cisplatin-vinorelbine, used for discovery and validation

	Signature discovery	Validation
**Description**	TCGA	Zhu et al.
**Accession #**	TCGA-LUAD[2]	GSE14814[5]
**Platform**	Illumina HiSeq 2000 (mRNA expression)<br>Illumina Infinium Human Methylation (HM450) array (DNA methylation)	Affymetrix Human Genome U133A
**Patients**	8	39
**Sample collection**	surgery	surgery
**Histological subtype**		
mixed	6	NA
acinar	1	9
papillary	NA	5
mucinous	NA	1
lepidic	NA	1
solid	NA	9
NOS	1	14
**Anatomic Site**		
Left-Upper	2	NA
Left-Lower	NA	NA
Right-Lower	2	NA
Right-Middle	1	NA
Right-Upper	3	NA
**Gender**		
Female	5	20
Male	3	19
**Tumor Stage (Pathological)**		
IA	NA	8
IB	1	14
II	NA	NA
IIA	3	11
IIB	1	6
IIIA	2	NA
IIIB	NA	NA
IV	1	NA
**Smoking Status**		
1	1	NA
2	NA	NA
3	4	NA
4	3	NA

Notes: NA = Not available, NOS = Not otherwise specified.
Smoking status: 1 = lifelong non-smoker (<100 cigarettes smoked in Lifetime), 2 = current smoker (includes daily smokers and non-daily smokers (or occasional smokers), 3 = current reformed smoker for > 15 years, 4 = current reformed smoker for ≤ 15 years.

Supplementary Table 4, related to Fig. 6, Supplementary Figure 8 Clinical and pathological features of lung squamous cell carcinoma patient cohorts treated with cisplatin-vinorelbine, used for discovery and validation.

	**Signature discovery**	**Validation**
**Description**	TCGA	Zhu et al.
**Accession #**	TCGA-LUSC[6]	GSE14814[5]
**Platform**	Illumina HiSeq 2000 (mRNA expression)	Affymetrix Human Genome U133A
	Illumina Infinium Human Methylation (HM450) array (DNA methylation)	
**Patients**	8	26
**Sample collection**	surgery	surgery
**Histological subtype**		
NOS	8	26
**Anatomic Site**		
Left-Upper	2	NA
Left-Lower	NA	NA
Right-Lower	4	NA
Right-Middle	1	NA
Right-Upper	1	NA
**Gender**		
Female	1	3
Male	7	23
**Tumor Stage (Pathological)**		
I	NA	13
IA	NA	NA
IB	2	NA
II	NA	13
IIA	1	NA
IIB	4	NA
IIIA	1	NA
IIIB	NA	NA
IV	NA	NA
**Smoking Status**		
1	NA	NA
2	NA	NA
3	2	NA
4	6	NA

Notes: NA = Not available, NOS = Not otherwise specified.

Smoking status: 1 = lifelong non-smoker (<100 cigarettes smoked in Lifetime), 2 = current smoker (includes daily smokers and non-daily smokers (or occasional smokers), 3 = current reformed smoker for > 15 years, 4 = current reformed smoker for ≤ 15 years.

Supplementary Table 5, related to Fig. 6, Supplementary Figure 8 Clinical and pathological features of colorectal adenocarcinoma patient cohorts treated with FOLFOX (folinic acid, fluorouracil, oxaliplatin), used for discovery and validation.

	Signature discovery	Validation
**Description**	TCGA	Marisa et al.
**Accession #**	TCGA-COAD[7]	GSE39582[8]
**Platform**	Illumina HiSeq 2000 (mRNA expression)<br>Illumina Infinium Human Methylation (HM450) array (DNA methylation)	Affymetrix Human Genome U133 Plus 2.0 Array
**Patients**	8	23
**Sample collection**	surgery	surgery
**Histological subtype**		
Ascending Colon	1	NA
Cecum	2	NA
Descending Colon	1	NA
Sigmoid Colon	3	NA
NA	1	NA
**Gender**		
Female	4	8
Male	4	15
**Tumor Stage (Pathological)**		
I	NA	NA
IA	NA	NA
IB	NA	NA
II	NA	NA
IIA	1	2
IIB	NA	1
III	1	NA
IIIA	1	3
IIIB	4	3
IIIC	1	3
IV	NA	11

Notes: NA = Not available.

Supplementary Table 6, Related to Supplementary Figure 7. Identified candidate pathways (carboplatin-paclitaxel treated LUAD, cisplatin-vinorelbine treated LUAD, cisplatin-vinorelbine treated LUSC, and FOLFOX (folinic acid, fluorouracil, oxaliplatin) treated COAD) readout, source, and contribution to cancer.

Cancer types & treatments	Candidate pathways	Readout	Source	Contribution to cancer
LUAD_CP	chemokine receptors bind chemokines	*CCL22*		promotes bone metastasis in lung cancer[9]
	mRNA splicing	*POLR2C*		therapeutic target in breast cancer[10]
	G alpha (s) signalling events	*PDE7A*		prognostic marker of lung cancer[11]
	intestinal immune network for IgA production	*CCR9*		prognostic marker of non-small cell lung cancer[12], etoposide resistance in prostate cancer[13], cisplatin resistance in breast[14] and ovarian[15] cancers
	metabolism of proteins	*CCT4*		therapeutic target in lung cancer[16]
	RNA degradation	*LSM7*		diagnostic marker of thyroid cancer[17]
	cell cycle mitotic	*FGFR1OP*		prognostic biomarker and therapeutic target in lung cancer[18]
LUAD_CV	metabolism of nucleotides	*DTYMK*		therapeutic target for LKB1-deficient lung cancer[19]
	actin Y	*ARPC1A*		novel marker of pancreatic cancer[20]
	ribosome	*RPLP2*		prognostic marker in gynecologic tumor[21] and in gastric cancer[22]
LUSC	cytokine-cytokine receptor interaction	*CCL11*		biomarker of ovarian cancer[23]
	neuroactive ligand-receptor interaction	*GABRA1*		DNA methylation markers in colorectal cancer[24]
	DNA repair	*ERCC1*		prognostic marker in prostate[25], and bladder[26] cancer
	SLC-mediated transmembrane transport	*SLC44A4*		novel target for prostate and pancreatic cancer[27]
	translation	*RPL14*		molecular marker for esophageal squamous cell carcinoma[28]
	transport of mature mRNA derived from an intron-containing transcript	*U2AF1*		contributes to cancer progression[29]
COAD	elongation and processing of capped transcripts	*SF3B3*		therapeutic target for ER-positive breast cancer[30]
	processing of capped intron containing pre mRNA	*PRPF6*		tumor marker in colon cancer[31]
	metabolism of protein	*PFDN1*		promotes epithelial-mesenchymal transition (EMT) and lung cancer progression[32]
	S phase	*CDC25B*		prognostic marker in non-small cell lung cancer[33]
	calcium signaling	*MYLK3*		biomarker in ovarian cancer[34]

Notes: LUAD_CP = lung adenocarcinoma treated with carboplatin and paclitaxel; LUAD_CV = lung adenocarcinoma treated with cisplatin and vinorelbine; LUSC = lung squamous cell carcinoma treated with cisplatin and vinorelbine; COAD = colon adenocarcinoma treated with FOLFOX (folinic acid, fluorouracil, oxaliplatin); Source (fourth column): readout in each pathway are represented as differentially expressed (pink), methylated (grey) and both differentially expressed and methylated (yellow).

Supplementary Discussion

To evaluate the clinical importance of these pathways, we have investigated potential therapeutic targeting of pathway genes using canSAR[35], a computational chemogenomic analysis, which connects molecular alterations to therapeutic targeting with approved or investigational drugs (or drug candidates for future clinical trials). In particular, we have discovered that *CXCR5* (*chemokine receptor* pathway) can be targeted by immunostimulant plerixafor, which has already shown promising result in phase I advanced pancreatic cancer clinical trial[36] and in cervical cancer[37]. A known driver *MAP2K1* (*immune network for IgA production* pathway) can be targeted by MEK (i.e., mitogen-activated protein kinase) inhibitors (i.e., trametinib, cobimetinib), which are known to improve disease course for several cancers including, KRAS mutated non-small cell lung cancer[38], BRAF-mutated melanoma[39], and KRAS/BRAF-mutated colorectal cancer[40]. Another interesting candidate is *PDE7B* (*G alpha (s) signalling events* pathway) can be targeted by xanthines (i.e., theophylline, dyphylline), which have been shown to attenuate tumor metastasis in melanoma[41, 42]. We anticipate that further investigation of the therapeutic targeting of the identified pathways can advance optimal treatment guidelines for patients with predisposition to carboplatin-paclitaxel resistance.

Supplementary References

1. Gu, Z., Gu, L., Eils, R., Schlesner, M. & Brors, B. circlize Implements and enhances circular visualization in R. *Bioinformatics* **30**, 2811-2812 (2014).
2. Comprehensive molecular profiling of lung adenocarcinoma. *Nature* **511**, 543-550 (2014).
3. Tang, H. *et al.* A 12-gene set predicts survival benefits from adjuvant chemotherapy in non-small cell lung cancer patients. *Clinical cancer research : an official journal of the American Association for Cancer Research* **19**, 1577-1586 (2013).
4. Love, M.I., Huber, W. & Anders, S. Moderated estimation of fold change and dispersion for RNA-seq data with DESeq2. *Genome Biology* **15**, 550 (2014).
5. Zhu, C.Q. *et al.* Prognostic and predictive gene signature for adjuvant chemotherapy in resected non-small-cell lung cancer. *Journal of clinical oncology : official journal of the American Society of Clinical Oncology* **28**, 4417-4424 (2010).
6. Comprehensive genomic characterization of squamous cell lung cancers. *Nature* **489**, 519-525 (2012).
7. Comprehensive molecular characterization of human colon and rectal cancer. *Nature* **487**, 330-337 (2012).
8. Marisa, L. *et al.* Gene expression classification of colon cancer into molecular subtypes: characterization, validation, and prognostic value. *PLoS medicine* **10**, e1001453 (2013).
9. Nakamura, E.S. *et al.* RANKL-induced CCL22/macrophage-derived chemokine produced from osteoclasts potentially promotes the bone metastasis of lung cancer expressing its receptor CCR4. *Clinical & experimental metastasis* **23**, 9-18 (2006).
10. Grinchuk, O.V. *et al.* Sense-antisense gene-pairs in breast cancer and associated pathological pathways. *Oncotarget* **6**, 42197-42221 (2015).

11. Beer, D.G. *et al.* Gene-expression profiles predict survival of patients with lung adenocarcinoma. *Nature medicine* **8**, 816-824 (2002).
12. Gupta, P. *et al.* CCR9/CCL25 expression in non-small cell lung cancer correlates with aggressive disease and mediates key steps of metastasis. *Oncotarget* **5**, 10170-10179 (2014).
13. Sharma, P.K. *et al.* CCR9 mediates PI3K/AKT-dependent antiapoptotic signals in prostate cancer cells and inhibition of CCR9-CCL25 interaction enhances the cytotoxic effects of etoposide. *International journal of cancer* **127**, 2020-2030 (2010).
14. Johnson-Holiday, C. *et al.* CCR9-CCL25 interactions promote cisplatin resistance in breast cancer cell through Akt activation in a PI3K-dependent and FAK-independent fashion. *World journal of surgical oncology* **9**, 46 (2011).
15. Johnson, E.L. *et al.* CCR9 interactions support ovarian cancer cell survival and resistance to cisplatin-induced apoptosis in a PI3K-dependent and FAK-independent fashion. *Journal of ovarian research* **3**, 15 (2010).
16. Vishnubhotla, P., Carr, A.C., Khaled, A., Bassiouni, R. & Khaled, A.R. CT20p as a therapeutic for lung cancer with elevated chaperonin containing TCP1 (CCT) expression levels. *Journal of Clinical Oncology* **35**, e23163-e23163 (2017).
17. Tomei, S. *et al.* A molecular computational model improves the preoperative diagnosis of thyroid nodules. *BMC cancer* **12**, 396 (2012).
18. Mano, Y. *et al.* Fibroblast growth factor receptor 1 oncogene partner as a novel prognostic biomarker and therapeutic target for lung cancer. *Cancer science* **98**, 1902-1913 (2007).
19. Liu, Y. *et al.* Metabolic and functional genomic studies identify deoxythymidylate kinase as a target in LKB1-mutant lung cancer. *Cancer discovery* **3**, 870-879 (2013).
20. Laurila, E., Savinainen, K., Kuuselo, R., Karhu, R. & Kallioniemi, A. Characterization of the 7q21-q22 amplicon identifies ARPC1A, a subunit of the Arp2/3 complex, as a regulator of cell migration and invasion in pancreatic cancer. *Genes, chromosomes & cancer* **48**, 330-339 (2009).
21. Artero-Castro, A. *et al.* Expression of the ribosomal proteins Rplp0, Rplp1, and Rplp2 in gynecologic tumors. *Human pathology* **42**, 194-203 (2011).
22. Zhang, Y.-Z. *et al.* Discovery and validation of prognostic markers in gastric cancer by genome-wide expression profiling. *World journal of gastroenterology: WJG* **17**, 1710 (2011).
23. Levina, V. *et al.* Role of eotaxin-1 signaling in ovarian cancer. *Clinical cancer research : an official journal of the American Association for Cancer Research* **15**, 2647-2656 (2009).
24. Lee, S. *et al.* Identification of GABRA1 and LAMA2 as new DNA methylation markers in colorectal cancer. *International journal of oncology* **40**, 889-898 (2012).
25. Jacobsen, F. *et al.* Increased ERCC1 expression is linked to chromosomal aberrations and adverse tumor biology in prostate cancer. *BMC cancer* **17**, 504 (2017).
26. Bellmunt, J. *et al.* Gene expression of ERCC1 as a novel prognostic marker in advanced bladder cancer patients receiving cisplatin-based chemotherapy. *Annals of Oncology* **18**, 522-528 (2007).
27. Mattie, M. *et al.* The discovery and preclinical development of ASG-5ME, an antibody–drug conjugate targeting SLC44A4-positive epithelial tumors including pancreatic and prostate cancer. *Molecular cancer therapeutics* **15**, 2679-2687 (2016).
28. Huang, X.P. *et al.* Alteration of RPL14 in squamous cell carcinomas and preneoplastic lesions of the esophagus. *Gene* **366**, 161-168 (2006).
29. Palangat, M. *et al.* The splicing factor U2AF1 contributes to cancer progression through a noncanonical role in translation regulation. *Genes & development* **33**, 482-497 (2019).
30. Gokmen-Polar, Y. *et al.* Expression levels of SF3B3 correlate with prognosis and endocrine resistance in estrogen receptor-positive breast cancer. *Modern pathology : an official journal of the United States and Canadian Academy of Pathology, Inc* **28**, 677-685 (2015).
31. Adler, A.S. *et al.* An integrative analysis of colon cancer identifies an essential function for PRPF6 in tumor growth. *Genes & development* **28**, 1068-1084 (2014).
32. Wang, D. *et al.* Prefoldin 1 promotes EMT and lung cancer progression by suppressing cyclin A expression. *Oncogene* **36**, 885 (2016).
33. Sasaki, H. *et al.* Expression of the cdc25B gene as a prognosis marker in non-small cell lung cancer. *Cancer letters* **173**, 187-192 (2001).

34. Phelps, D.L. *et al.* Methylation of MYLK3 gene promoter region: a biomarker to stratify surgical care in ovarian cancer in a multicentre study. *British journal of cancer* **116**, 1287-1293 (2017).
35. Tym, J.E. *et al.* canSAR: an updated cancer research and drug discovery knowledgebase. *Nucleic Acids Res* **44**, D938-943 (2016).
36. Torphy, R.J., Zhu, Y. & Schulick, R.D. Immunotherapy for pancreatic cancer: Barriers and breakthroughs. *Annals of gastroenterological surgery* **2**, 274-281 (2018).
37. Chaudary, N. *et al.* Plerixafor Improves Primary Tumor Response and Reduces Metastases in Cervical Cancer Treated with Radio-Chemotherapy. *Clinical Cancer Research* **23**, 1242-1249 (2017).
38. Janne, P.A. *et al.* Selumetinib plus docetaxel for KRAS-mutant advanced non-small-cell lung cancer: a randomised, multicentre, placebo-controlled, phase 2 study. *The Lancet. Oncology* **14**, 38-47 (2013).
39. Flaherty, K.T. *et al.* Improved survival with MEK inhibition in BRAF-mutated melanoma. *The New England journal of medicine* **367**, 107-114 (2012).
40. Yeh, J.J. *et al.* KRAS/BRAF mutation status and ERK1/2 activation as biomarkers for MEK1/2 inhibitor therapy in colorectal cancer. *Molecular Cancer Therapeutics* **8**, 834-843 (2009).
41. Lentini, A. *et al.* Preclinical evaluation of the antineoplastic efficacy of 7-(2-hydroxyethyl)theophylline on melanoma cancer cells. *Melanoma research* **22**, 133-139 (2012).
42. Sawa, T., Wu, J., Akaike, T. & Maeda, H. Tumor-targeting chemotherapy by a xanthine oxidase-polymer conjugate that generates oxygen-free radicals in tumor tissue. *Cancer research* **60**, 666-671 (2000).
