# Supplementary material for: pathCHEMO, a generalizable computational framework uncovers molecular pathways of chemoresistance in lung adenocarcinoma
Source: Commun Biol. 2019 Sep 6;2:334. doi: 10.1038/s42003-019-0572-6 (PMC6731276; doi:10.1038/s42003-019-0572-6)
Supplement: Supplementary file 2 — Description of additional supplementary files [file 42003_2019_572_MOESM2_ESM.docx]

**Description of Additional Supplementary Files**

**Supplementary Datasets**

**File Name:** Supplementary Dataset 1

**Description: (1A)** Treatment response (carboplatin-paclitaxel) differential expression signature, comparing poor response and favorable response patients (p < 0.05). **(1B)** Treatment response (carboplatin-paclitaxel) differential methylation signature, comparing poor response and favorable response patients (p < 0.05).

**File Name:** Supplementary Dataset 2

**Description: (2A)** Treatment response (carboplatin-paclitaxel in lung adenocarcinoma) composite expression pathway signature, comparing poor response and favorable response patients, with sign of the analysis, NES, p-value, and genes in the leading edge indicated. **(2B)** Treatment response (carboplatin-paclitaxel in lung adenocarcinoma) composite methylation pathway signature, comparing poor response and favorable response patients, with sign of the analysis, NES, p-value, and genes in the leading edge indicated.

**File Name:** Supplementary Dataset 3

**Description:** **(3A)** Overlaps between carboplatin-paclitaxel treated LUAD, and cisplatin-vinorelbine treated LUAD, cisplatin-vinorelbine treated LUSC, FOLFOX (folinic acid, fluorouracil, oxaliplatin) treated COAD candidate transcriptomic pathways. **(3B)** Overlaps between carboplatin-paclitaxel treated LUAD, and cisplatin-vinorelbine treated LUAD, cisplatin-vinorelbine treated LUSC, FOLFOX (folinic acid, fluorouracil, oxaliplatin) treated COAD candidate epigenomic pathways. **(3C)** Overlaps between cisplatin-vinorelbine treated LUAD, and carboplatin-paclitaxel treated LUAD, cisplatin-vinorelbine treated LUSC, FOLFOX (folinic acid, fluorouracil, oxaliplatin) treated COAD candidate transcriptomic pathways. **(3D)** Overlaps between cisplatin-vinorelbine treated LUAD, and carboplatin-paclitaxel treated LUAD, cisplatin-vinorelbine treated LUSC, FOLFOX (folinic acid, fluorouracil, oxaliplatin) treated COAD candidate epigenomic pathways. **(3E)** Overlaps between cisplatin-vinorelbine treated LUSC, and carboplatin-paclitaxel treated LUAD, cisplatin-vinorelbine treated LUAD, FOLFOX (folinic acid, fluorouracil, oxaliplatin) treated COAD candidate transcriptomic pathways. **(3F)** Overlaps between cisplatin-vinorelbine treated LUSC, and carboplatin-paclitaxel treated LUAD, cisplatin-vinorelbine treated LUAD, FOLFOX (folinic acid, fluorouracil, oxaliplatin) treated COAD candidate epigenomic pathways. **(3G)** Overlaps between FOLFOX (folinic acid, fluorouracil, oxaliplatin) treated COAD, and carboplatin-paclitaxel treated LUAD, cisplatin-vinorelbine treated LUAD, cisplatin-vinorelbine treated LUSC candidate transcriptomic pathways. **(3H)** Overlaps between FOLFOX (folinic acid, fluorouracil, oxaliplatin) treated COAD, and carboplatin-paclitaxel treated LUAD, cisplatin-vinorelbine treated LUAD, cisplatin-vinorelbine treated LUSC candidate epigenomic pathways.
