# Supplementary material for: pathCHEMO, a generalizable computational framework uncovers molecular pathways of chemoresistance in lung adenocarcinoma
Source: Commun Biol. 2019 Sep 6;2:334. doi: 10.1038/s42003-019-0572-6 (PMC6731276; doi:10.1038/s42003-019-0572-6)
Supplement: Supplementary file 7 — Peer Review File [file 42003_2019_572_MOESM7_ESM.pdf]

Editorial Note: This manuscript has been previously reviewed at another journal. This document only contains reviewer comments and rebuttal letters for versions considered at *Communications Biology* .

REVIEWERS' COMMENTS:

Reviewer #1 (Remarks to the Author):

Authors have adequately addressed my concerns. I have no issues with it and recommend accept by Communications Biology.

Reviewer #2 (Remarks to the Author):

I think the authors have adresed all my previous concerns appropriately. I do think the manuscript has improved substantially and I am happy to see it in press.

REVIEWERS' COMMENTS:<br><br>Reviewer #1 (Remarks to the Author):<br><br>Authors have adequately addressed my concerns. I have no issues with it and recommend accept by Communications Biology.<br><br><br><br>Reviewer #2 (Remarks to the Author):<br><br>I think the authors have adresed all my previous concerns appropriately. I do think the manuscript has improved substantially and I am happy to see it in press.	We are genuinely thankful to the Reviewers for their favorable evaluation of our manuscript
